# A participatory expert-based risk assessment to inform zoonotic disease surveillance in Cameroon: the case of Ebola virus disease and Rift Valley fever

**DOI:** 10.1186/s42522-026-00232-5

**Published:** 2026-08-07

**Authors:** Inès Sopbué Kamguem, Nathalie Kirschvink, Nicolas Antoine-Moussiaux, Florence De Longueville, Cécile Ingrid Djuikoue, Noel K. Joseph, Sophie Molia, Abel Wade, Catherine Linard

**Affiliations:** 1 Institute of Life, Earth and Environment (ILEE), University of Namur, Namur, 5000 Belgium; 2 Namur Research Institute for Life Sciences (NARILIS), University of Namur, Namur, 5000 Belgium; 3 Fundamental and Applied Research for Animals & Health (FARAH), University of Liège, Liège, 4000 Belgium; 4 Department of Public Health Sciences, Faculty of Medicine, University of Liège, Liège, 4000 Belgium; 5 CIRAD, UMR ASTRE, Montpellier, F-34398 France; 6 ASTRE, Université de Montpellier, CIRAD, INRAE, Montpellier, France; 7 Laboratoire National Vétérinaire (LANAVET), Yaoundé, Cameroon

**Keywords:** Risk assessment, Participatory epidemiology, Rift Valley fever, Ebola virus disease, One Health, Multi-criteria decision analysis, Cameroon

## Abstract

**Background:**

Viral haemorrhagic fevers (VHFs) are a group of zoonotic diseases caused by RNA viruses, including pathogens such as Ebola virus, Marburg virus, Lassa virus, and Rift Valley fever virus. Previous studies have identified Cameroon as a high-risk country, due to favourable ecoclimatic conditions and the presence of susceptible mammals and vectors that have been confirmed positive for viruses causing Rift Valley fever (RVF) and Ebola virus disease (EVD). Although evidence exists of RVF virus circulation in animals and serological reactivity to Ebola-related viruses among humans in Cameroon, the country has not officially documented epidemics of RVF or EVD. The objective of this study is to provide novel, context-refined knowledge of perceived risk factors and temporal patterns of RVF and EVD emergence in Cameroon, where epidemiological data on these two diseases are limited, with RVF explored through the livestock component and its early transmission dynamics in animal hosts, and zoonotic spillover to humans for EVD.

**Methods:**

The present work proposes a risk assessment of RVF and EVD emergence through the development of a One Health integrated risk assessment tool for identifying perceived high-risk periods and key risk factors. A mixed, semi-quantitative approach involving expert semi-structured interviews, and a monthly calendar was used for data collection. Expert-based risk evaluation was then compared to risk factors derived from the scientific literature.

**Results:**

For RVF, the wettest months seem to pose the highest risk, with increased inter-animal transmission during livestock transhumance. Key spatial determinants identified by experts included rainfall, host density, reservoirs, vectors, and livestock movements. For EVD spillover risk, human activities and behaviours, land cover, and host density were also perceived as critical. Convergences and divergences regarding expert-based and literature-based risk factors for RVF and EVD were observed, with variations concerning respectively livestock trade and socio-cultural practices linked to livelihood and forest-based subsistence. The distinction is likely attributable to the differing approaches of contextualization and generalization employed respectively by the two sources of weighting.

**Conclusions:**

This study shows that, even in regions with no documented disease history, an integrated approach combining expert knowledge and literature-based evidence enables meaningful perceived risk assessment, generates novel and context-specific information, and supports the refinement of preventive strategies. The participatory visual tool introduced allows identification and quantification of putative zoonotic risk factors and their temporal dynamics, informing risk mapping, management, and communication.

**Supplementary Information:**

The online version contains supplementary material available at https://doi.org/10.1186/s42522-026-00232-5.

## Background

Over the last three to four decades, the central region of Africa has seen an increase in documented occurrence of zoonotic diseases, particularly viral haemorrhagic fevers (VHFs), including mosquito-borne arboviruses such as yellow fever and dengue [1, 2], alongside recurrent documented outbreaks of non-vector-borne VHFs, particularly Ebola virus disease (EVD) and Marburg virus disease [3, 4]. Viral haemorrhagic fevers present acute hazards to human and animal health, marked by high morbidity and mortality rates, efficient human-to-human transmission, and the potential to trigger large-scale epidemics and global pandemics [5]. Cameroon is considered to be at high risk for several VHFs, particularly EVD and Rift Valley fever (RVF) [6, 7], while no human clinical case of EVD and no outbreaks of RVF has been reported yet [8]. Ebola virus disease, caused specifically by Ebola virus (species *Zaire ebolavirus*), is included in the list of national priority zoonotic diseases under surveillance in Cameroon [9, 10] due to the high transmissibility of symptomatic individuals [11], the geographical proximity of Cameroon to countries that have already experienced multiple epidemic outbreaks [6, 12], and it’s devastating impact [13]. Mpouam et al. (2024) highlight that, although not a national priority for epidemiological surveillance, RVF is recognized as a critical animal disease in Cameroon, impacting livestock production, economy, trade, and public health, especially in high-density livestock regions [8]. Owing to its neglected status, coupled with the existence of serological evidence of viral circulation in livestock and humans, authors such as Sado et al. (2022) assert that outbreaks of RVF may occur in Cameroon without being detected [14]. However, current surveillance strategies lack context-specific risk assessments. Cameroon spans highly contrasted ecological zones, ranging from Sahelian and Sudanian landscapes in the north to humid forest ecosystems in the south [15]. These zones differ not only in vegetation and wildlife assemblages, but also in rainfall regime, with a predominant unimodal rainy season in the north and bimodal rainfall in much of the south [16]. Livestock production represents a key component of the national economy, particularly in the northern Regions (Adamawa, North, and Far North), where pastoral and agropastoral systems predominate [17, 18]. These northern Regions are characterized by high densities of cattle and small ruminants and seasonal transhumance [19, 20]. Such ecological heterogeneity is likely to influence the seasonality and spatial distribution of zoonotic emergence risk. To our knowledge, no study has comprehensively assessed the ecological, climatic, and socio-economic determinants of RVF and EVD emergence in Cameroon, nor the associated risk periods, especially in the context of limited epidemiological data. In addition, surveillance planning in Cameroon has yet to systematically incorporate participatory inputs from in-country experts who may possess critical frontline knowledge of emerging disease risks. To address this gap, this study elicits local expert knowledge through a participatory risk assessment approach to identify priority perceived high risk periods and key risk factors for targeted, risk-based surveillance. RVF and EVD were selected because they represent an interesting juxtaposition. RVF is a vector-borne disease for which ecological drivers and seasonal dynamics are relatively well described. The related literature highlights a potential prior human case and serological evidence in Cameroon, which suggest possible viral circulation within some reservoir hosts and vectors [7, 21–23]. By contrast, EVD presents a more uncertain ecological profile: no clear spillover event has been documented and the literature reports only putative reservoir hosts. This contrast made it possible to explore how expert elicitation performs across diseases with different levels of ecological and epidemiological certainty.

Risk-based approaches overcome the limitations of traditional, fragmented strategies by integrating surveillance, coordinated interventions, and proactive risk mitigation, rather than relying on reactive outbreak responses that are more costly [24, 25]. In contexts where a disease has never been clinically documented but is recognized as a potential emerging threat, integrating non-empirical local knowledge into formal risk analyses is essential to better estimate and anticipate risk and to strengthen prevention and control strategies [26]. Emphasizing interdisciplinary collaboration and community engagement, risk-based approaches leverage new tools and technologies to address the complex interplay of factors driving zoonotic disease emergence, ultimately enhancing mitigation efforts [27, 28]. In data and resource-limited environments, participatory disease risk assessment has proven especially useful to gather data on potential risk factors for emerging animal and zoonotic diseases [29, 30]. By drawing on local knowledge, it facilitates the design of disease prevention and control programs that are both acceptable to their stakeholders and effective [29, 31]. Wirsiy et al. [32] emphasized that successful prevention requires understanding putative risk factors, as this will be useful to develop intervention measures targeting communities at risk [32].

A participatory risk assessment framework was developed in this work to overcome the limited availability of health data needed to assess the risk of emergence and transmission of EVD and RVF in Cameroon. It combines methods from both participatory epidemiology (PE) [33] and multicriteria decision analysis (MCDA) [34] to collect stakeholders’ perceptions regarding high-risk periods and the main spatial risk factors driving the emergence of these diseases in the Cameroonian context through a semi-quantitative process. A risk assessment tool was adapted from the model developed by Catley et al. (2002) during an investigation into chronic wasting disease in southern Sudanese cattle, which employed a participatory method known as the “seasonal calendar” to capture local perceptions of seasonal variations in cattle diseases, disease vectors, intermediate hosts and rainfall [35]. In the present study, these last elements were replaced by georeferenceable (mappable) risk factors—termed spatial risk factors—defined by Stevens et al. (2013) within the spatial MCDA framework for occurrence of highly pathogenic avian influenza virus H5N1 in domestic poultry in Asia [36]. Furthermore, the representation of seasons by Catley et al. (2002) was refined into the twelve calendar months to allow for a more fine-scale temporal analysis. The objective of this study was to acquire a more comprehensive understanding of the periods and the drivers contributing to the potential zoonotic transmission of EVD (from animals to humans), caused by the species *Zaire ebolavirus*. It also aimed to identify those that may play a role in the potential epizootic emergence of RVF in livestock (domestic ruminants). This was done by developing an integrated zoonotic risk assessment tool for identifying perceived high-risk periods and key spatially relevant risk factors driving the emergence of these diseases in the Cameroonian context. In this paper, “emergence” refers to the appearance of a disease in a population, or to a rapid increase in the incidence or geographic extent of a disease [37]. The findings of this study may inform risk-based surveillance and prevention strategies of these two VHFs, as well as providing a critical foundation for disease risk mapping, supporting the identification of high-risk areas and guiding risk managers in strategically determining where and when to concentrate their efforts and allocate resources effectively.

## Methods

## Participatory data collection

### Study design

A semi-quantitative framework was developed by combining individual semi-structured interviews with Cameroonian disease experts, monthly calendar diagramming [33, 38], and spatial risk factors weighting through a participatory epidemiology methodology [30].

The semi-quantitative risk assessment focused on:

- Zoonotic spillover risk of EVD;
- Risk of RVF emergence in domestic ruminants.

Interview guides (Additional file 1) included open-ended questions grouped into two themes: temporal risk variation and risk factor identification. The guides were prepared in French and pre-tested with four respondents to refine unclear or confusing questions. Pre-test data were not used for data analysis. Each interview had two parts: one for RVF and one for EVD. All interviews were conducted and audio-recorded in French with participants’ consent. Interviews were transcribed in French, and relevant study materials, verbatim quotations and narrative summaries of participants’ views were translated into English during manuscript preparation, with care taken to preserve the original meaning of participants’ responses.

### Expert selection

A stakeholder identification and categorisation exercise was undertaken by a team of four Cameroonian interviewers and key informants to systematically identify and visualise the full spectrum of actors involved in the prevention and control of VHFs in Cameroon. Rather than applying a formal stakeholder mapping framework (e.g., power-interest grid or influence-impact matrix), the exercise focused on categorising and visually representing stakeholders using flowchart-style diagrams according to sector (human health, animal health, and wildlife), institutional type (governmental, non-governmental, public, and private), and administrative level (national, regional, and local). This process was used to ensure broad representation of relevant institutions and perspectives in the expert selection process. The selected actors included representatives of ministries, international organisations active in Cameroon (such as WHO, USAID and AFROHUN), academic staff from universities, health professionals, veterinarians, but also researchers with peer-reviewed publications related to the diseases of interest. The national institutions included the Ministry of Scientific Research and Innovation; the Ministry of Livestock, Fisheries and Animal Industries; the Ministry of Public Health; the Ministry of Forestry and Wildlife; the One Health platform within the Prime Minister’s Office; the National Veterinary Laboratory (LANAVET); the Research Centre for Emerging and Re-emerging Diseases (CREMER); and the National Veterinary Association of Cameroon (ONVC).

Eligibility criteria prioritised individuals holding positions within Cameroon’s national zoonoses surveillance and control systems, across both public and private sectors, and ideally who had carried out activities related to these diseases. Eligible experts required a relevant expertise in fields such as epidemiology, human medicine, veterinary medicine, microbiology, entomology, public health, environmental and wildlife sciences, and a minimum of one year’s experience in disease prevention and control to accurately assess seasonal variations in risk factors. Each expert completed a form (Additional file 2) designed to assess key criteria reflecting their expertise in the relevant diseases. Experts were recruited through snowball sampling until sample adequacy was achieved according to the principles of information power [39] and thematic saturation [40]. They were selected for the relevance of their expertise to the study objective, particularly their direct involvement in zoonoses management, surveillance, animal health, wildlife, and One Health activities in Cameroon. Recruitment proceeded through professional referral until additional participants no longer substantially expanded the diversity of sectoral and institutional profiles represented in the panel, and the interviews consistently yielded recurrent rather than novel risk factors. On this basis, the sample was considered adequate for the aims of the study.

Following the identification of experts considered relevant to the study, participation in the interviews was proposed to them on a voluntary basis, allowing each person the opportunity to either accept or decline the invitation freely. In total, 22 experts were interviewed, including 15 from the animal health sector, 6 from the human health sector, and 1 from the wildlife sector. This distribution reflects the experts’ willingness to participate, and their availability during the data collection period. Further characteristics of the interviewed experts, including professional background and disease-specific expertise are presented in Fig. 2.

### Data collection

Of the 29 Cameroonian disease experts invited for interviews, 22 consented to participate in the study. Participants were purposively selected to ensure diversity across the main sectors, institution types, and administrative levels involved in VHF prevention and control in Cameroon. The aim was not statistical representativeness, but in-depth qualitative insight from key experts identified through the stakeholder identification process. Semi-structured interviews were conducted from February to March 2024.

**Fig. 1:**
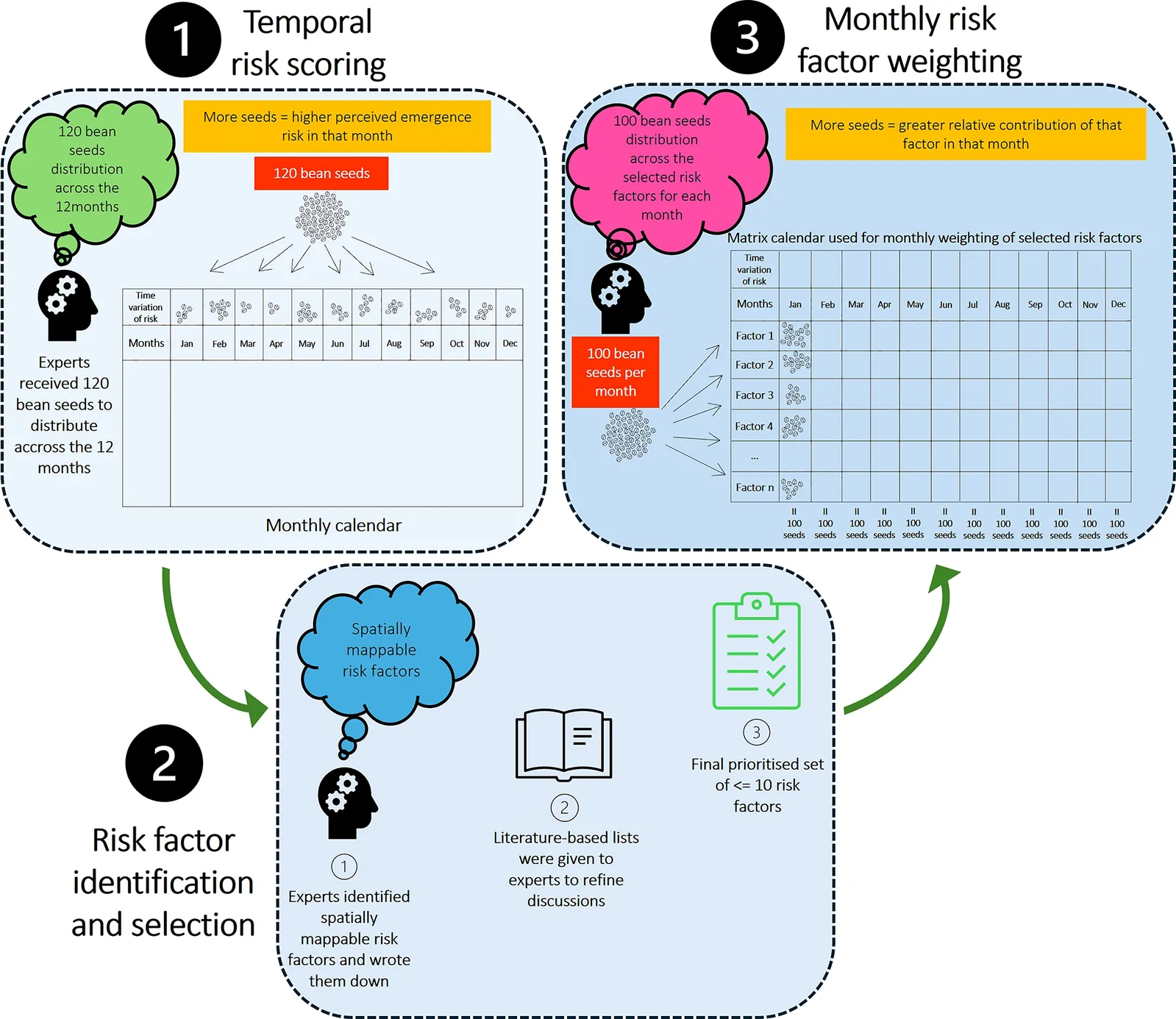
Schematic representation of the proportional piling procedure used during expert interviews

For each disease, the interviews were conducted in two distinct stages, each of which involved proportional piling [29, 33]:

- **Assessing time variation of risk**: First, experts were asked to carry out proportional piling using 120 bean seeds, which they had to distribute over the 12 months of the year to reflect the fluctuation in emergence risk throughout the year (risk scores). This was performed on the first row of the monthly calendar as shown in Fig. 1.
- **Risk factor identification and weighting**: Secondly, experts were asked to list spatially mappable risk factors. To this end, the experts were asked to identify the factors that help elucidate why a particular area is more susceptible to the emergence of the disease compared to another. Following this step, they were asked to refine the factors pointed out using literature-based lists and to prioritize a final set of ten or fewer. These lists stem from a scoping review of the literature, which identified spatially relevant determinants for the transmission of these diseases in the tropical rainforest of Central Africa [12]. A 12-month matrix calendar (January–December) was drawn on a large sheet, with months on the x-axis and risk factors on the y-axis (Fig. 1). Experts assigned weighted scores to each risk factor, reflecting its relative contribution to disease onset on a given month [34]. Using proportional piling, respondents distributed 100 bean seeds per month, allocating more seeds to factors with higher transmission risk (Additional file 3). Experts verified and adjusted final scores as needed.

## Data analysis

Descriptive and multivariate analyses of proportional piling data identified key trends in perceived risk variation and risk factors throughout the year.

### Expert corpus description

We first described experts according to the following characteristics: profession, disease knowledge, level of intervention in the health pyramid, sector of activity, and gender. Frequencies of each characteristic among the experts were calculated and plotted.

### Assessment of the variation in risk over time

The annual perceived risk variation curves were designed by computing for each disease, the averages of the number of seeds allocated by experts to different months of the year through proportional piling (risk scores).

### Key risk factors identification and expert weights calculation

Bar charts were generated by computing the number of times a risk factor was identified by experts. Heatmaps were constructed by calculating, for each month of the year, the average number of seeds allocated to each risk factor by all experts during the proportional piling process (weighting).

For each expert, the proportional weight assigned to a given risk factor for a given month was calculated as:

$$\:{w}_{i,j,m}=\frac{{s}_{i,j,m}}{100}$$

Where ***w***_***i, j,m***_ is the weight assigned by expert ***j*** to factor ***i*** in month ***m***, and **s**_***i, j,m***_ is the number of seeds allocated to that factor. Because each expert distributed 100 seeds across the selected factors, the sum of weights across factors equalled 1 for each expert and month.

For each factor and month, expert weights were then averaged across all experts who responded for that disease. Under this consideration rule, risk factors not selected by a given expert were treated as though that expert had assigned them a score of zero:

$$\:{\boldsymbol{w}}_{\boldsymbol{i},\boldsymbol{m}}=\frac{1}{E}\sum\limits_{j=1}^{E}{w}_{i,j,m}$$

Where ***w***_***i, m***_ is the final weight of factor ***i*** for month ***m***, and ***E*** is the total number of experts who responded for that disease.

Frequency of risk factor identification and average expert weight were treated as related but distinct indicators: the former captured how consistently a factor was selected across experts, whereas the latter reflected its mean relative importance across the full expert panel, based on proportional piling scores.

The concordance between experts’ weightings was then assessed by visually evaluating their variation using box plots. The data used consisted of the number of seeds allocated by each expert to key risk factors throughout the months of the year.

### Comparative analysis of expert weightings with those derived from the literature

For comparison purpose, we assigned a literature-based weight using the number of scientific articles mentioning each risk factor in a recent scoping review on spatial determinants for VHFs in the tropical rainforest of Central Africa [12] (Additional file 4). We used a scoping review approach because it allowed inclusion of studies with diverse designs and samples, which was appropriate for capturing the broad range of spatial determinants reported for these diseases. Search terms were “risk factor”, “viral haemorrhagic fevers”, and “Central Africa”. The review focused on countries included in the tropical and subtropical moist broadleaf forest ecoregion of Central Africa, based on the map of Battistella [41], hereafter referred to as the “African tropical moist forest”. The literature-based weighting was intended as a semi-quantitative comparative approach, designed to reflect the relative prominence of risk factors in the available literature. For each risk factor, we counted the number of studies in the scoping review that identified that factor as spatially relevant to disease emergence, and these counts were then normalised to generate relative literature-based weights across factors. Similar frequency-count approaches have been used in systematic [42] and scoping reviews [43] to summarise the relative prominence of reported determinants when studies are heterogeneous and not directly comparable. The weights attributed by the experts were calculated as the average of the weights assigned to each risk factor over the 12 months of the year. Graphs were then constructed to compare the weights obtained from these two different sources. A scatter plot was generated to assess the distribution of weights derived from the literature in relation to those obtained from expert weightings. This analysis enabled the evaluation of the normality and linearity of these distributions, followed by the calculation of the Spearman’s rank correlation coefficient, which quantifies the degree of agreement between the risk factors rankings assigned by experts and those established in the literature. In addition, to quantify divergences between expert and literature weights, individual relative discrepancies were calculated as the absolute difference between expert and literature weights, divided by the literature weight and expressed as a percentage, providing a reference baseline to assess how much expert assessments deviated from published estimates according to the following formula:

$$\eqalign{ & {\rm{Relative}}\>{\rm{discrepancy}}\>\left( \% \right) \cr & = {{|{W_{{\rm{expert}}}} - {W_{{\rm{literature}}}}|} \over {{W_{{\rm{literature}}}}}} \times \>100 \cr}$$

To complement the relative discrepancy analysis, Bland-Altman plots were generated for both diseases to provide a complementary visual assessment of agreement and quantitative differences between expert- and literature-derived weights [44, 45]; in these plots, positive differences indicate higher expert-derived weights, whereas negative differences indicate higher literature-derived weights.

The statistical analyses were performed using R software version 4.4.0, accessed through the RStudio interface (version 2024.12.1–563) [46].

### Qualitative data analysis

For a deeper analysis of the rationale behind the choice of each risk factor by experts, qualitative analysis was performed. Data were pseudonymized by replacing the names of the experts with coded identifiers based on profession (VET for veterinarians, MED for medical doctors, RES for researcher, PHP for public health professional, and FOW for forestry and wildlife professional). Pseudonymization involves processing personal data to prevent direct attribution to an individual without supplementary information. It replaces explicit identifiers (e.g., name) with indirect ones (e.g. classification numbers) while remaining reversible, unlike anonymization [47]. Pseudonymized records were securely stored. The thematic content analysis was the method employed to analyse the interviews [48]. The transcription (transforming oral discourse into text) was manually completed using Microsoft Word, while ensuring the anonymity of the participants. During the thematization process (which was also done manually), the information was organised by themes and sub-themes, with a descriptor (code) assigned to each theme. The analysis of themes and sub-themes drew inspiration both from those established in advance based on the literature, as well as from the identification of new themes that emerged from the various discourses. Through this method, we aimed to faithfully and objectively represent what the interviewees expressed.

## Ethics statement

The study was conducted under official research authorization granted by the Ministry of Livestock, Fisheries and Animal Industries through the National Veterinary Laboratory of Cameroon (N/Ref.: L 24–068/LANAVET/DG/CAB). All experts received explanations regarding the study and signed an informed consent form. Participation was voluntary, with privacy and confidentiality ensured per the Belgian law (30 July 2018) on data protection. Interviews were recorded with experts’ consent. Anonymity and data protection have been ensured in compliance with the General Regulation on the Protection of Personal Data to maintain the confidentiality of participants during data processing and analysis.

## Results

## Expert corpus description

**Fig. 2:**
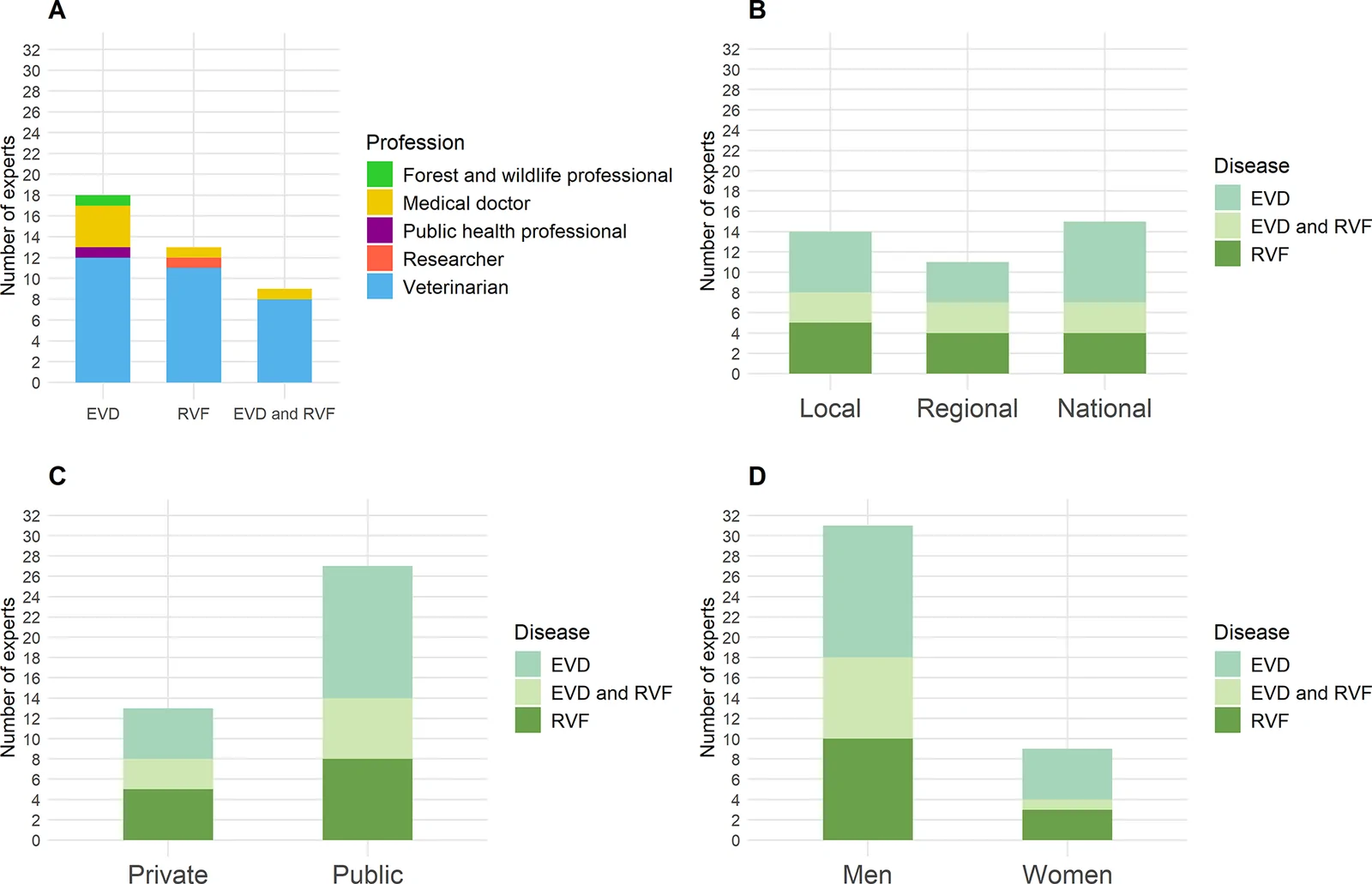
Distribution of experts by: **(A)** disease knowledge and profession; **(B)** level of decision-making; **(C)** sector; **(D)** gender

The main characteristics of the interviewed experts are shown in Fig. 2. Overall, 22 unique experts participated in the study. Because some experts were interviewed on both disease topics, the disease-specific counts are overlapping rather than mutually exclusive (Additional file 5): 18 experts answered questions on EVD, 13 on RVF, and 9 of these experts contributed to both interview modules.

With respect to their professions, most of the experts in this study were veterinarians (68%). Fe

wer medical doctors were knowledgeable about RVF transmission in livestock, and most of the respondents who answered all the questions on RVF were veterinarians (Fig. 2A). Most of the experts held positions at the national decision-making level (Fig. 2B) and were from the public sector (Fig. 2C). The expert panel was predominantly male (Fig. 2D).

Because the results of the experts’ self-assessments were highly inconsistent, with individuals of similar academic and professional backgrounds assigning themselves markedly different scores, this criterion was excluded from the analysis. The self-evaluation was considered too subjective to be used as a reliable metric.

## Results of the integrated zoonotic risk assessment tool

### Assessment of the variation in risk over time

#### Risk variation of Rift Valley fever emerging in livestock

**Fig. 3:**
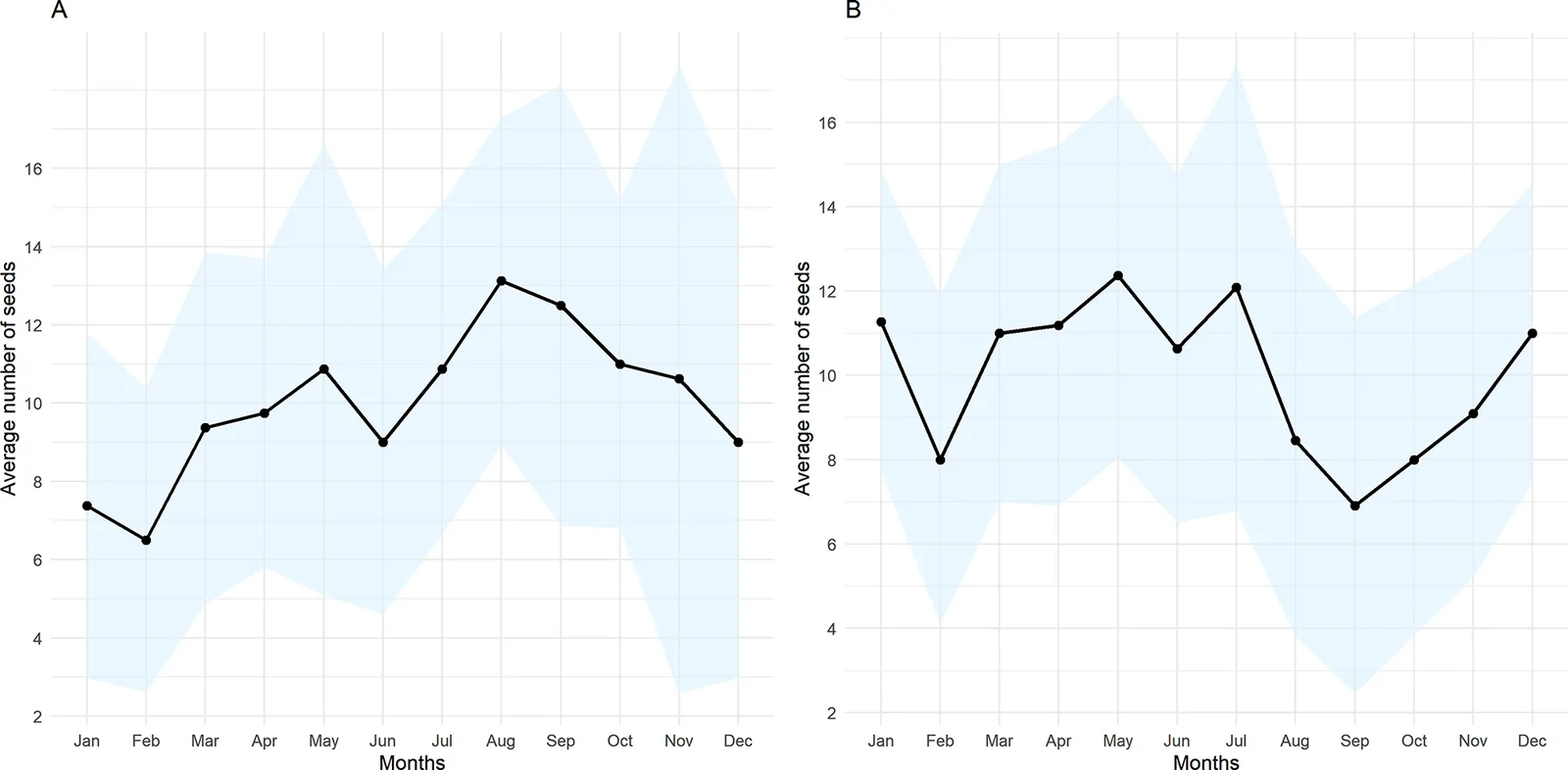
Average expert’s weighting of the perceived risk per month for: **(A)** RVF and **(B)** EVD. The black curves plot the average number of seeds per month. The light-blue bands show the 95% confidence interval, visualizing the variability around the mean

The curve representing the average perceived risk weighting shows a risk peak in August and September (Fig. 3A). According to the respondents, these months represent the period with the highest average rainfall in the northern regions of Cameroon and to the period when there is a high probability of flooding. Flooding has been identified as a factor favouring the hatching of *Aedes* and *Culex* mosquito eggs carrying the RVF virus. One month after the period of heavy rainfall, the risk of RVF emergence is highest, as mosquito eggs hatch, said a participating researcher specialised in medical and veterinary entomology research:

*October and November mark the end of rainy season and beginning of transhumance, which is characterized by livestock movements and increased contact between domestic herds and wildlife at watering points in the savannah with mosquito pullulation. I conducted a study that confirmed the presence of the virus in captive monkeys in Cameroon. Additionally, recent research has shown that when farmers reported contact between wildlife and their herds, a high seroprevalence of the virus was found in the livestock. Specifically, this contact involved antelopes in the savannah areas of northern Cameroon.*

All experts agreed that the period of greatest risk was the off-season, at the end of the heaviest rains. As a result of climate change, the end of the heaviest rainfall was somewhat heterogeneous from one expert to another.

#### Risk variation of Ebola virus disease spillover to humans

In this context, with no EVD epidemics nor clinical cases reported, experts unanimously agreed that establishing a distinct seasonality for the risk remains challenging. However, the analysis of the main determinants and their temporal variations revealed that May, July, December, and January correspond to periods of highest risk, based on average risk weights (Fig. 3B). The period encompassing all these months match the authorised hunting period for big game, running from December to July (hunting starts in May-June) as stated by the wildlife expert consulted. Thus, the period of significantly reduced risk coincides with the closure of legal hunting seasons in designated hunting zones. July corresponds to the holiday period when road travels are frequent —particularly along routes connecting the East, Centre, Littoral and West Regions. People are accustomed to eating cooked bushmeat bought at the roadside during the journey, as well as uncooked meat to take back. May and July fall during the rainy season where various trees bear fruit that attract both animals and humans. This is associated with a greater likelihood of contact between wildlife and humans. Similarly, in the southern part of the country, agricultural activities are more important during the rainy season, and the risk of contact is greater when accessing agricultural areas within or bordering forested landscapes. December and January (early January) have been mentioned as high-risk periods due to the intensive sale, handling and consumption of bushmeat because of the end-of-year festivities throughout the country. Crop harvest also attracts animals and wildlife that come to feed on grains and farm products, creating proximity and more contacts among them, favoring hunting activities. However, experts underscored that the risk remains pervasive throughout the year, driven by the ongoing practices of small game hunting (hunting of smaller animals like rodents, bats, hares), along with the trade and consumption of bushmeat nationwide.

### Key spatial risk factors identified by experts

**Fig. 4:**
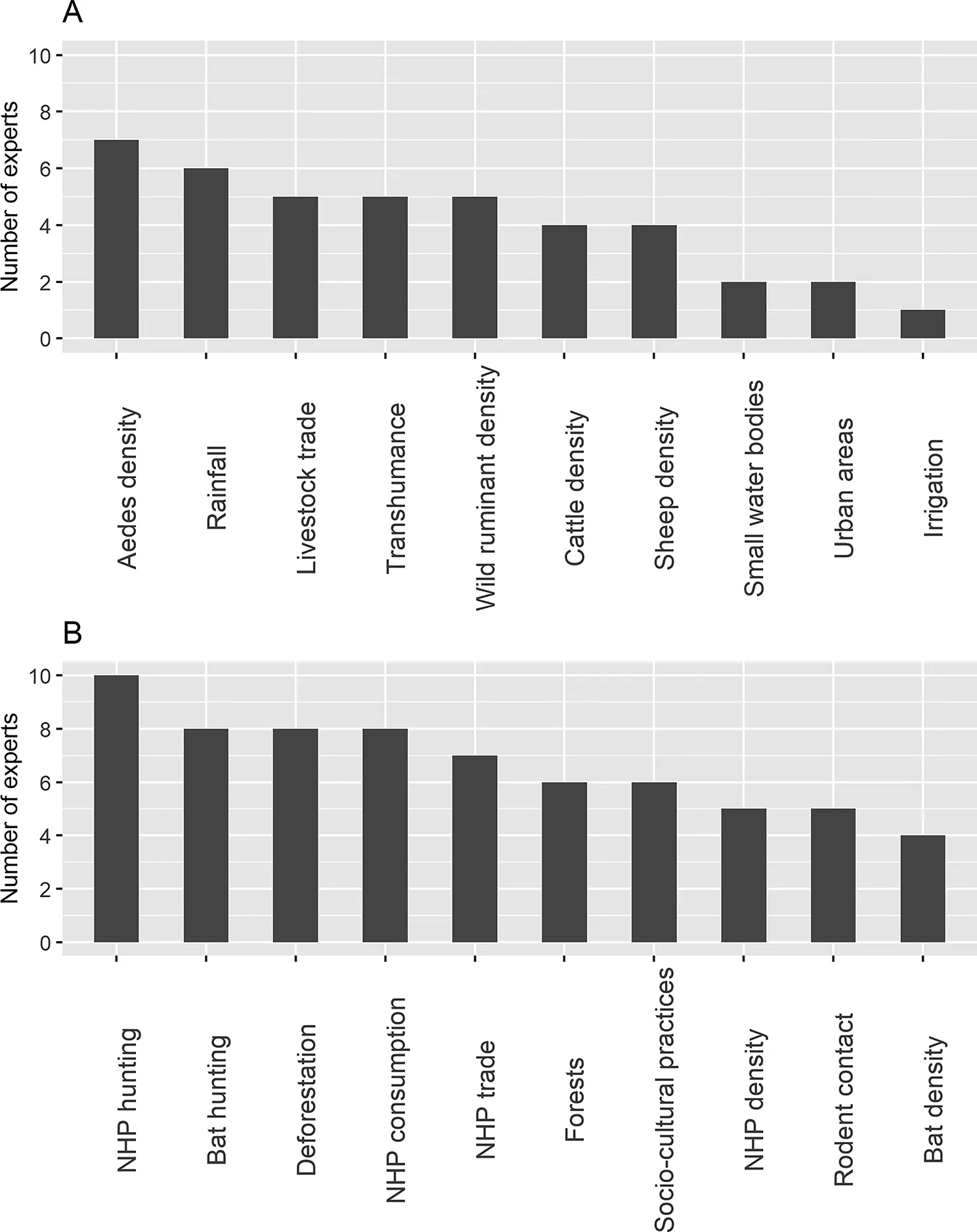
Ordered bar charts of identified risk factors for: **(A)** RVF and **(B)** EVD. The x-axis denotes the risk factors, and the y-axis indicates the number of respondents who identified each risk factor. NHP = non-human primates

**Fig. 5:**
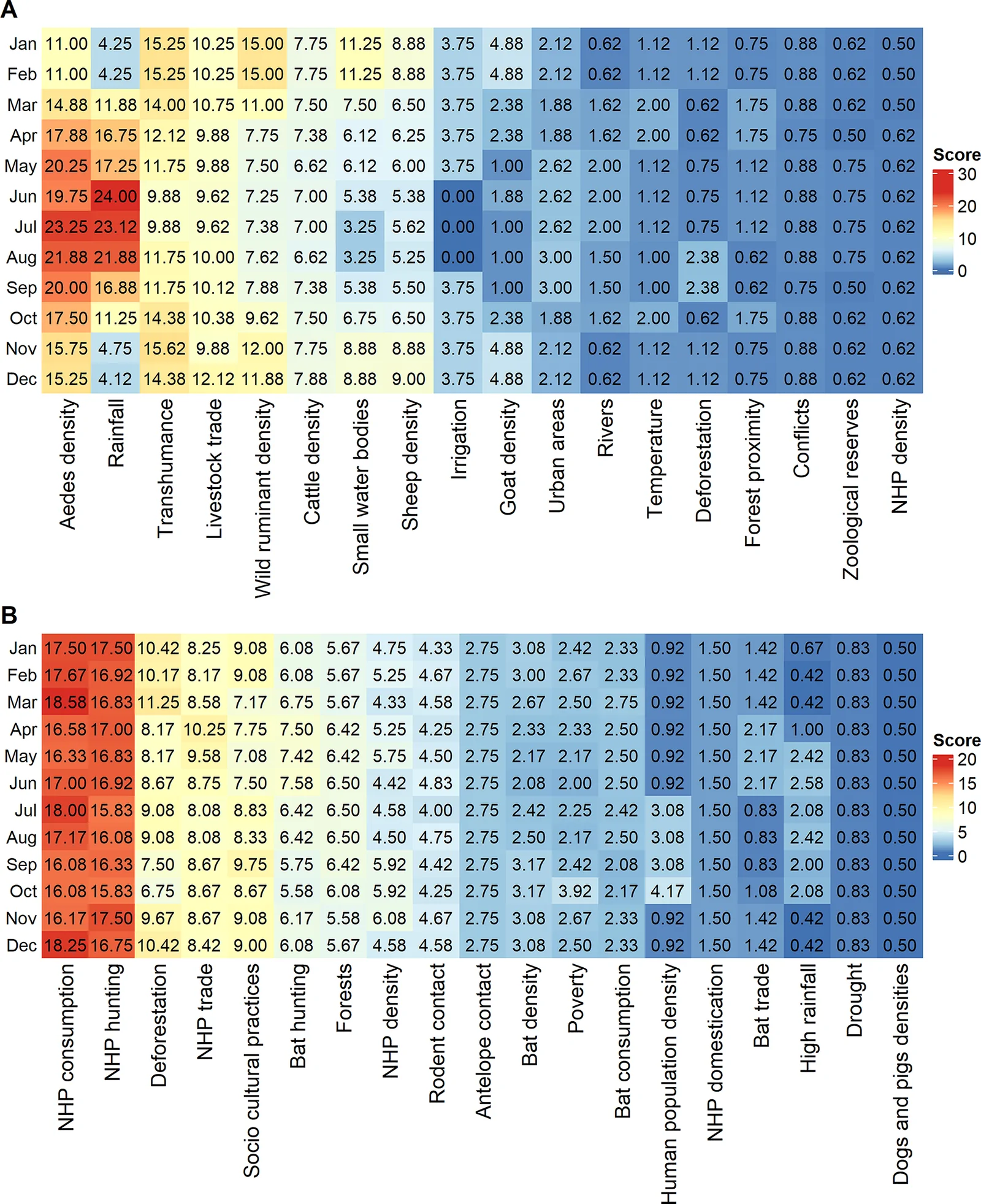
Heatmaps of risk factors resulting from weighting process for: **(A)** RVF and **(B)** EVD. The x-axis represents the risk factors, and the y-axis indicates the months of the year. Each row denotes the average numbers of seeds allocated to each risk factor by all experts during the proportional piling process

Key spatial risk factors were identified by combining two different representations. Initially, the selection was based on the ten risk factors identified by the highest number of experts, as illustrated by the bar charts, where the x-axis denotes the risk factors, and the y-axis indicates the number of respondents who identified each risk factor (Fig. 4). Secondly, the 10 factors with the highest average weights from proportional piling over the 12 months of the year (Fig. 5) were selected. The key drivers that emerged were those common to both methods. A comprehensive table (Additional file 6) is presented to define each risk factor identified through the scoping review of the literature as well as through expert interviews conducted in the field.

According to these figures, the four major perceived risk factors identified by experts conducive for RVF emergence in livestock are in decreasing order of importance: mosquito density (genus *Aedes* and to a lesser extent those of the genus *Culex*), rainfall, transhumance and livestock trade. Those related to EVD are in similar order: non-human primates (NHP) hunting, trade and consumption, deforestation, and socio-cultural practices linked to livelihood and forest-based subsistence.

Regarding herd movements identified as RVF main risk factors, a participating veterinary expert and sub-regional representative of an international health organization, emphasized that the threat does not arise from the movements per se, but rather from their lack of regulation and oversight:

*Porous borders promote transhumance and facilitate broader commercial livestock movements, with livestock moving across Nigeria, Cameroon, and Sudan, while herds from Chad traverse Bénoué and Faro National Parks before reaching the Adamawa Region. Wild and domestic animals often share routes and water sources, increasing regulatory challenges. The lack or poor enforcement of livestock management policies, particularly in transport, calls for a reassessment of health certification. Some herds bypass official routes to evade checkpoints, aided by complicit officials who create unauthorized passages.*

The interviews also revealed water as the common denominator in RVF transmission, as mosquitoes’ life cycle depends on it. Seasonal transhumance and nomadism are likewise driven by water and pasture availability. An expert highlighted this, citing a study in the Sudano-Sahelian zone near Lagdo town, where cattle had higher RVF seroprevalence due to irrigated farming. Heavy rainfall, particularly in August and September (in northern Cameroon), has intensified flooding in recent years, potentially linked to climate change he was stating. The same expert added that watering points likely play a significant role in maintaining the virus year-round, potentially triggering outbreaks during the dry season when awareness of the risk may be insufficient. Some PCR-positive samples have been identified in cattle and sheep, which indicate the presence of viral genetic material. However, no confirmatory tests (such as virus isolation or sequencing) have yet confirmed active viral presence.

Transhumance is more important in May and November. These are the months when the transhumance herds return home (May) and go out to graze (November), bringing them into closer contact with wildlife and other domestic herds from countries such as Sudan, Chad, Niger and Nigeria, as they share the same grazing and watering areas (periods during which *Aedes* and *Culex* swarm at the water’s edge). November is also the month when temporary water bodies are newly formed and still well-filled following the recent rains, particularly in the *Logone River* floodplain in the Far North Region. According to experts, this refers to « Yaere ». These are temporary water accumulated through the river *Logone* and constitutes an ecological interface that facilitates transmission by promoting contact between livestock, wildlife, and mosquito vectors. The experts say that transhumance follows a seasonal north-south pattern in Cameroon. During the dry season in the northern Regions (November to April), herders from the Far North, North, and Adamawa Regions move southward toward the Adamawa Plateau and forest-savannah transition zones (e.g. localities such as Tignère, Ngaoundal, and Tibati) in search of pasture and water. With the onset of the rainy season in the same Regions (May), they return northward as resources regenerate. According to participating experts, these movements may also extend across borders into Chad, the Central African Republic, or Nigeria, depending on environmental and security conditions.

As far as EVD is concerned, experts agreed that hunting, processing, transport and trade of bushmeat (both raw and cooked), pose the highest risk, followed by its consumption. Notably, bushmeat sold in markets may also originate from other countries. However, they stated that it is crucial to first assess the probability that the bushmeat consumed is contaminated with Ebola virus. Hunting of gorillas and chimpanzees is legally prohibited, as they are fully protected species, and their commercial trade is forbidden. However, their meat is prized by local populations, exacerbating poaching, a significant perceived risk factor. According to them, porcupine and rat meats are widely consumed delicacies, particularly in rural areas, while antelope, monkeys, and great apes are also considered coveted treats with high demand, especially in urban centres such as Yaoundé, Douala and Bertoua. A participant public health authority of the South Region underscored the socio-cultural practices linked to livelihood and forest-based subsistence as a behavioural driver. This was explained by the fact that in forested areas, communities, particularly in the South Region, maintain close ties with primates due to cultural traditions, rituals, and hunting practices that foster direct contact. Many, including Pygmies, spend several months in the deep forest, relying on bushmeat (especially bats and great apes) as a key food source before returning to their campsites.

According to several experts, deforestation is predominant in January. An expert from the Ministry of Forests and Fauna attributed the greatest weight to that factor as conducive to EVD spillover, corroborating the words of a participating veterinary and a public health expert:

*Anthropogenic factors such as deforestation for major infrastructure projects (e.g.*,* Memve’ele and Lom-Pangar dams*,* several deep-water ports)*,* and logging disrupt wildlife habitats*,* forcing animal displacement and increasing human-wildlife interactions. Contrary to common assumptions*,* risk is not limited to dense forests. In northern savannah and Sahelian regions*,* drought drives wildlife toward human settlements in search of water and food*,* heightening contact at shared water sources. Additionally*,* these regions contain several hunting zones*,* particularly in the Bénoué*,* Mayo Louti*,* and Faro divisions*,* which are notable for their high primate populations*,* further increasing the potential for human-wildlife interactions.*

In southern Cameroon, the hunting of non-human primates was also identified as a major factor during the month of January due to easy access to the forest for hunters (dry season).

### Concordance among experts regarding key risk factors weighting

**Fig. 6:**
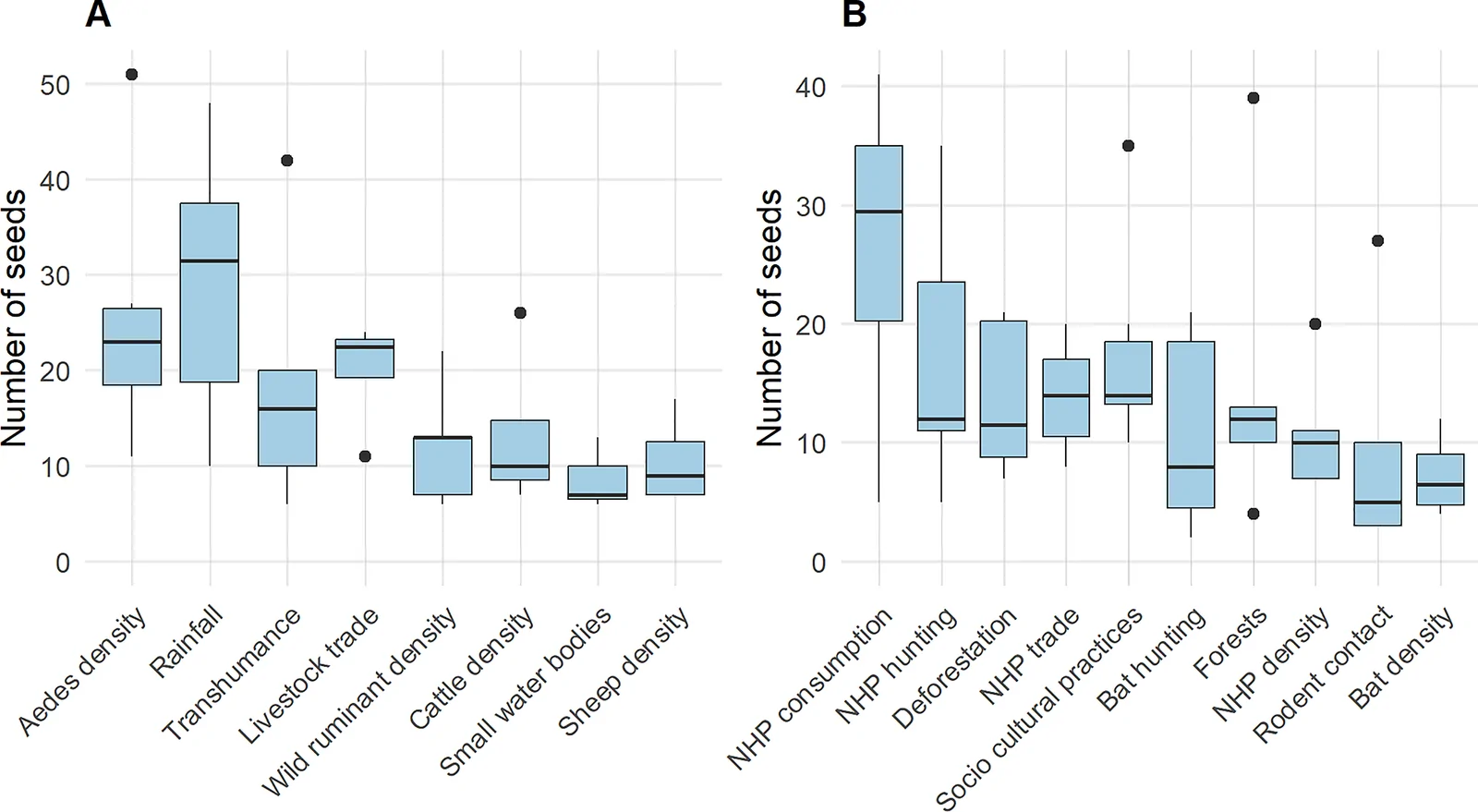
Box plots showing expert weighting alignment in August for RVF key perceived risk factors **(A)** and in July for EVD key perceived risk factors **(B)**

Boxplots for each perceived high-risk month assess the concordance between the weights assigned by the experts for key risk factors (Fig. 6). The short-length boxes of risk factors show that the variability in weights is low, which translates into greater accordance between experts.

The trends in seed allocation varied according to both risk factors and the months of the year. Overall, these figures illustrate that expert weightings exhibit a great convergence regarding risk factors related to water availability (for RVF), as well as those associated with year-end festivities and holiday periods in the case of EVD-related risk factors.

For RVF, most experts showed greater consensus regarding the relative importance of small water bodies during the month associated with the highest risk (August) (Fig. 6A). Similarly, stronger alignment was observed concerning the significance of *Aedes* density in May, small water bodies in November and December, as well as the critical role of livestock trade in April, May, June, July, August and September (Additional file 7).

Box plot analyses revealed varying degrees of concordance among experts regarding the weighting of factors influencing EVD spillover risk. Notably, in July (one of the months identified as posing the highest risk) there was stronger alignment among experts on the significance of NHP density as a contributing factor (Fig. 6B). Weights exhibited closely matching values regarding deforestation in October, NHP trade in April, bat density in April, May and June (Additional file 8).

## Comparison of risk factor weighting: expert assessments vs. scientific literature

To ensure consistency in the terminology used to designate risk factors across both the scientific literature and expert assessments, we aggregated certain factors. For instance, in the case of hunting, individual factors such as the NHP hunting, bat hunting, rodent and antelope hunting, assessed separately by experts, were merged to facilitate comparison with the broader and more general category of bushmeat hunting commonly found in the literature [12]. Other terminological adjustments have been made for comparison purposes. The final list of risk factors considered, and their associated weightings are presented in Tables 1 and 2.

**Table 1:** Weights assigned to RVF risk factors by literature and experts

Category	Risk factors	Weights	
		**Literature**	**Experts**
Environmental variables	Heavy rainfall and flooding	0,12	0,13
	Temperature	0,03	0,01
	El Nino Southern Oscillation (ENSO)	0,01	/
	Climate change	0,01	/
	Rivers and streams	0,06	0,01
	Small water bodies	0,09	0,07
	Vegetation index & proximity to the forest	0,09	0,01
	Loss of forest cover	0,02	0,01
	Rainfed croplands	0,01	/
	Irrigation	0,08	0,03
	Ponds	0,03	/
	Elevation	0,04	/
Animal distribution and movements	*Aedes* density	0,10	0,17
	Cattle density	0,05	0,07
	Sheep density	0,07	0,07
	Other domestic animals (e.g. Goats)	0,01	0,03
	Livestock movements (transhumance & nomadism)	0,06	0,13
	Livestock trade	0,01	0,10
	Wild ruminant density	0,04	0,11
	Non-human primate density	/	0,01
Human variables	Urban areas	0,02	0,02
	Socio-political crisis (conflicts)	0,01	0,01
	Saving water in small dams	0,01	/

**Table 2:** Weights assigned to EVD risk factors by literature and experts

Category	Risk factors	Weights	
		**Literature**	**Experts**
Environmental variables	Temperature	0,10	/
	Forest cover	0,08	0,07
	Rainfall	0,03	0,01
	Elevation	0,03	/
	Cropland mosaics	0,02	/
	Evapotranspiration	0,02	/
	Drought	/	0,01
Animal distribution and movements	Non-human primate density	0,15	0,05
	Bat density	0,07	0,03
	Dogs and pigs densities	0,01	0,01
Human variables	Bushmeat hunting	0,14	0,28
	Bushmeat trade	0,04	0,10
	Bushmeat consumption	0,10	0,24
	Deforestation	0,04	0,09
	Non-human primate domestication	/	0,02
	Human population density	0,07	0,02
	Poverty	0,06	0,03
	Socio-cultural practices	0,03	0,08
	Education level	0,02	/

**Fig. 7:**
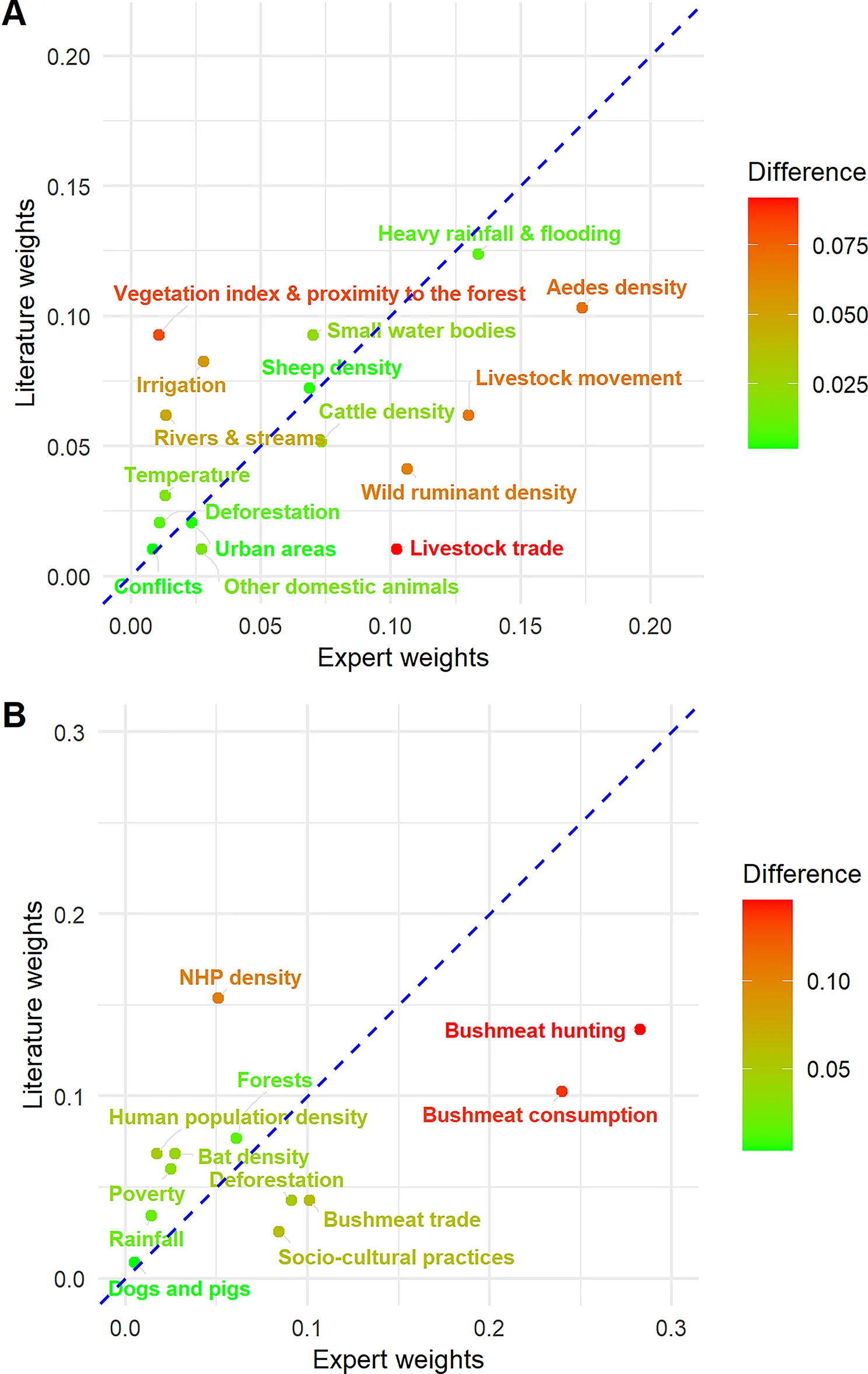
Scatter plot showing experts vs. literature weights for **(A)** RVF and **(B)** EVD drivers. The 1:1 dashed line serves as a reference and represents perfect concordance between the weights from the literature and those from experts, where their values are equal for each data point (risk factor)

**Fig. 8:**
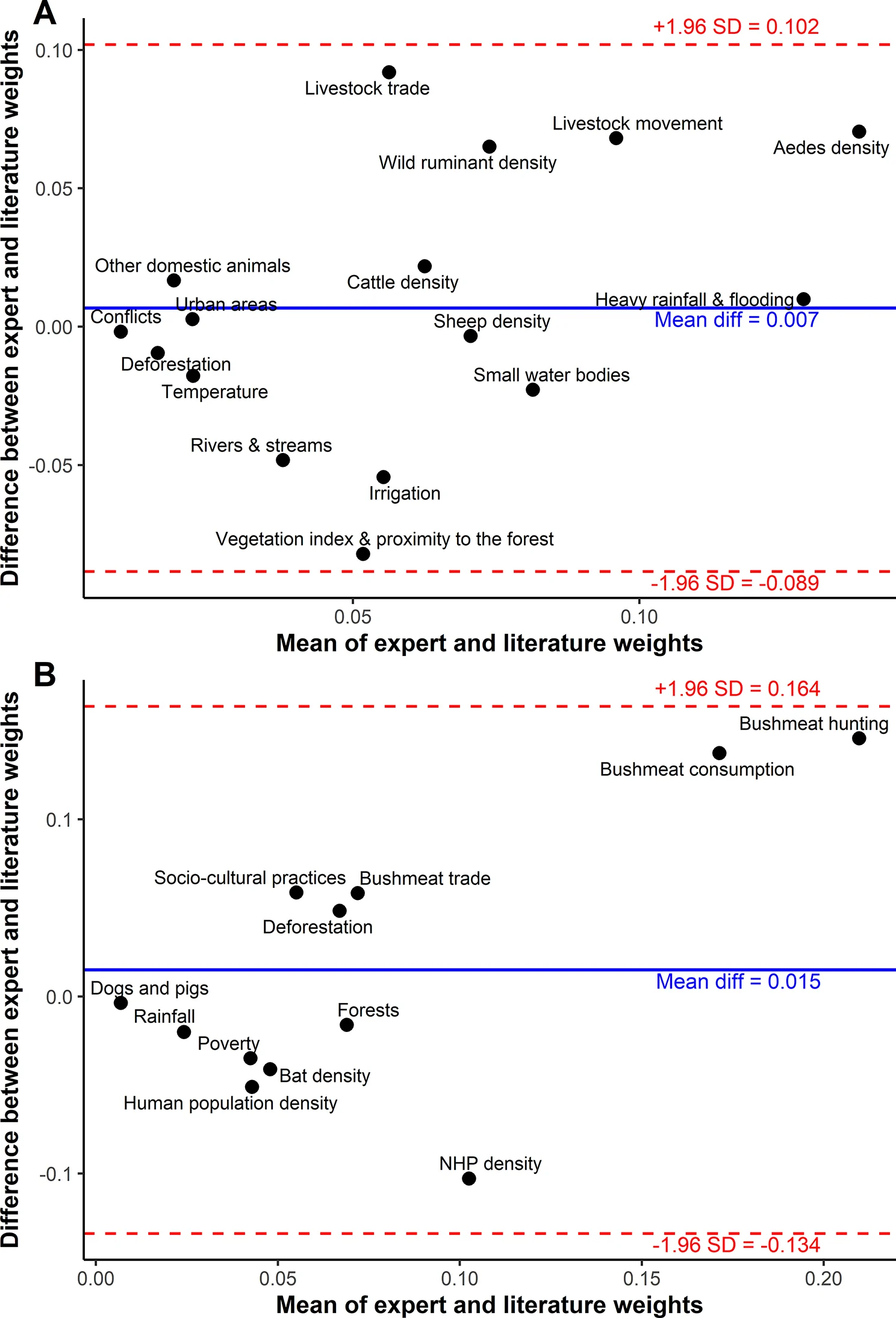
Bland–Altman plots comparing expert- and literature-derived weights for **(A)** RVF and **(B)** EVD. For each risk factor, the x-axis shows the mean of the expert- and literature-derived weights, and the y-axis shows the difference calculated as expert weight minus literature weight. The solid blue line represents the mean difference between expert- and literature-derived weights, while the red dashed lines indicate the 95% limits of agreement (mean difference ± 1.96 standard deviations). Positive values indicate risk factors for which experts assigned higher weights than the literature, whereas negative values indicate risk factors for which literature-derived weights were higher than expert-derived weights

Regarding RVF, the graph in Fig. 7A highlights that, according to both experts and the scientific literature, heavy rainfall and *Aedes* density are the most heavily weighted factors, appearing to be essential for the emergence of this disease in livestock in Cameroon. The difference in weighting between the two sources is not high for heavy rainfall, hence its green colour and the proximity of this factor to the 1:1 line. Figure 7A illustrates similar weight values assigned by both sources for the following factors: cattle and sheep densities, the presence of other domestic animals such as goats, heavy rainfall and flooding, temperature, loss of forest cover, urban areas, small water bodies, and socio-political crises. Experts cite the presence of non-human primates as a factor facilitating the transmission of RVF to livestock, although it did not emerge from the literature. However, they do not regard certain factors identified in the literature—such as water storage in small dams, the El Niño Southern Oscillation, rainfed croplands, ponds and elevation—as essential (Table 1). To assess the agreement between the weightings of both sources, and consequently the rankings they assign to each of the risk factors, the Spearman’s rank correlation coefficient (**ρ** = 0.43, p-value = 0.098) appeared moderate and not significant. With the aim of identifying the risk factors for which there are similarities and divergences between the two weighting methods, individual relative discrepancies were calculated (Additional file 9), which highlighted that livestock trade is the primary factor on which weighting divergence occurs, experts giving a greater weight. The corresponding Bland-Altman plot in Fig. 8A also indicated a slight overall positive mean difference between expert- and literature-derived weights (positive differences indicate higher expert-derived weights, whereas negative differences indicate higher literature-derived weights). The most pronounced positive deviations concerned livestock trade, livestock movement, *Aedes* density, and wildlife, whereas the most marked negative deviation was observed for vegetation index and proximity to the forest, followed by irrigation and rivers and streams.

By assigning weights to EVD spillover determinants identified in the literature, it becomes evident that the highest-weighted risk factors, according to both the literature and expert assessments, are hunting and the consumption of bushmeat (Fig. 7B). However, while NHP density is regarded as the most predominant risk factor in the literature, the presence of other domestic animals, such as dogs and pigs, is assigned the lowest weight across both weighting approaches. Certain factors, including the non-human primate domestication and drought, emerged during expert interviews but were absent from the literature. Conversely, factors such as altitude, temperature, cropland mosaics, education level, and evapotranspiration—recognised in the literature—were not deemed critical to Ebola virus spillover by experts in the Cameroonian context (Table 2). While forest cover, rainfall, and the influence of domestic animals (such as dogs and pigs) show relative alignment (Fig. 7B), the Spearman’s rank correlation coefficient (**ρ** = 0.44, p-value = 0.154) indicates only moderate agreement. This shows a certain common trend, but there are also discrepancies regarding the relative importance of socio-cultural practices linked to livelihood and forest-based subsistence (mainly), bushmeat consumption and trade for EVD spillover (Additional file 11), experts considering them more important than the literature does. The corresponding Bland-Altman plot (Fig. 8B) showed a slight overall positive mean difference between expert- and literature-derived weights. The most pronounced positive deviations concerned bushmeat hunting and bushmeat consumption, whereas non-human primate density showed the strongest negative deviation.

## Discussion

This study assessed the relative importance of putative risk factors for the emergence of RVF in livestock and EVD in humans in Cameroon by integrating semi-structured interviews with a semi-quantitative risk assessment tool, drawing on the expertise of local zoonotic disease experts. The findings elucidate both seasonal and non-seasonal perceived risk factors of these zoonoses, with expert consensus identifying 8–10 principal risk factors, several of which were also reported in the literature. From both sources of knowledge, key risk factors conducive for RVF emergence in livestock are mosquito density (genus *Aedes* and to a lesser extent those of the genus *Culex*), rainfall, transhumance, cattle and sheep densities, small water bodies, irrigation. Those related to EVD are non-human primates (NHP) density, hunting, trade and consumption, bat hunting and forest cover. However, previously undocumented determinants also emerged. Notably, the domestication of NHP as pets, absent from the prior scoping review [12], was reported by interlocutors, raising concerns for public health, animal welfare, and conservation. Additionally, experts reframed drought not merely as a lack of rainfall but as an ecological driver for EVD spillover, bringing wildlife closer to human settlements and increasing contact at shared water sources. For RVF, interactions among livestock, bats, and NHP surfaced as a potential risk factor, though less extensively documented in the literature [12]. Experts tended to assign greater importance to *Aedes* density than *Culex* density, likely because *Aedes* mosquitoes are perceived as the main initiators of RVF transmission in domestic ruminants, particularly following heavy rainfall and flooding of breeding sites. In contrast, *Culex* mosquitoes are generally considered important secondary vectors that contribute mainly to the amplification and spread of outbreaks once transmission has already been established.

In a setting with no documented history of disease but recognized emergence potential, this study offers valuable understanding of the spatially relevant perceived risk factors and periods of heightened risk for EVD spillover and RVF emergence in domestic ruminant populations. In such contexts, integrating local knowledge into formal risk analyses is essential for anticipating risk and strengthening prevention and control strategies [26]. The comparative analysis of expert assessments and existing literature suggested areas of convergence as well as divergence in the perceived significance of various risk factors, underscoring the complexity of these disease’s epidemiology. However, the observed Spearman’s rank correlation coefficients were moderate and not statistically significant for either disease, so these patterns should be interpreted as indicative of partial similarity in ranking, and not evidence of strong agreement. For RVF, both sources highlighted heavy rainfall and *Aedes* density as key factors, though experts assign greater significance to livestock trade. This difference may reflect the local context, where livestock markets, which form a network for livestock trade, are recognized as key hotspots for the emergence and transmission of various infectious diseases, including RVF [17]. In a similar context to Cameroon (silent circulation of the virus with no declared clinical cases), in Malawi, livestock in areas receiving 1001–1500 mm of rainfall had a significantly higher risk of RVF seropositivity (OR: 2.47; 95% CI: 1.14–5.37), underscoring high rainfall as a key priority risk factor for virus emergence and transmission [49]. In Sudan, heavy rainfall was strongly and significantly associated with RVF seropositivity in one-humped camels (OR: 5.36; 95% CI: 1.46–19.66), highlighting it as a major risk factor requiring priority attention [50]. Moreover, these results are broadly consistent with studies from affected countries such as Madagascar and Kenya, which identify key risk factors such as mosquito density, cattle trade, livestock densities (cattle and sheep), presence of small water bodies, and irrigation practices as critical contributors to RVF transmission in animals [51, 52].

Regarding EVD, hunting and bushmeat consumption consistently ranked as the most critical risk factors. However, experts attributed greater importance to socio-cultural practices linked to livelihood and forest-based subsistence than the literature, potentially due to its underrepresentation in peer-reviewed studies. This divergence may arise from contextual versus generalised approaches, with experts drawing on region-specific socio-cultural dynamics through lived experience and field observations, while the literature relies on broader models. Comparatively, bushmeat handling and consumption—particularly of duikers (OR = 5.57, 95% CI = 1.23–25.17), rodents (OR = 3.48, 95% CI = 0.70–17.23), and NHP (OR = 2.57, 95% CI = 0.30–22.27)—along with Batwa tribe membership linked to primate hunting, were strongly associated with Ebola virus seropositivity in Uganda [53], supporting the importance, in our results, of bushmeat hunting and consumption linked to socio-cultural practices as key perceived drivers of Ebola virus spillover. Moreover, in Ugandan forest communities, direct contact with primates yielded an OR = 6.53 (95% CI = 2.28–27.73) for individuals living in forest fragments, highlighting increased exposure linked to forested environments [54]. This further supports the inclusion of forest cover among the major perceived factors influencing the potential spillover of Ebola virus to humans.

A limitation of this comparison is that the literature-based weighting approach relies on the frequency with which a risk factor is reported in the literature. Such frequency does not necessarily reflect its biological importance. Some factors may be overrepresented because they have been more intensively studied, whereas others may appear less prominent due to unequal research effort, publication biases, or the heterogeneity of available studies. These weights should therefore be interpreted as reflecting the visibility of risk factors in the reviewed literature rather than their true epidemiological importance.

The observed divergence between expert-derived and literature-based assessments of the importance of certain risk factor may be interpreted as reflecting the complementarity between literature-based knowledge and experiential knowledge. The complementarity between scientific literature and field-based expert knowledge is best understood through the lens of knowledge integration—conceived as the critical synthesis of diverse perspectives to construct a more comprehensive understanding—where recognising and interpreting differences, rather than seeking consensus, fosters coherence by valuing the distinct contributions, assumptions, and contextual specificities of each knowledge source [55]. This study highlights the added value of integrating expert opinion, particularly in settings such as sub-Saharan Africa [56] where local realities, informal practices, and context-specific drivers may not be fully reflected in the scientific literature. In the Cameroonian context, where both EVD and RVF pose complex and evolving threats, the integration of these two knowledge systems may provide a more nuanced and contextually grounded understanding of disease emergence. These findings underscore the importance of incorporating local expertise into risk assessment frameworks, especially in regions where data may be sparse, and where lived experience can provide critical information for anticipating and mitigating zoonotic threats [23].

The integration of semi-quantitative methods enhances risk assessment by enabling measurable estimates critical for spatial indices, while qualitative inputs elucidate causal and contextual dimensions inaccessible to purely quantitative analysis; together, mixed-methods synthesis yields a more comprehensive and nuanced understanding of risk [57]. Grounded in the One Health framework, this research integrates diverse experts’ perspectives and fosters strong stakeholder engagement. Notably, all interviewed experts expressed willingness for being informed about the study’s findings and future developments, particularly regarding the potential adaptation of the tool for other diseases. Their insights also underscored the overlooked nature of RVF in Cameroon and offered a pragmatic comprehension into certain critical local realities. These include constraints in surveillance capacity, logistical challenges in livestock and wildlife monitoring, and gaps in community-level risk awareness as well as limited awareness among some health professionals, particularly regarding RVF. Such knowledge revealed operational bottlenecks that surpass external perspectives, underscoring the value of participatory approaches in operationalising One Health. As policymakers and funders increasingly advocate for stakeholder-driven research, early engagement with national health authorities enhances both the credibility and real-world applicability of findings [55], facilitating their integration into national policies [23]. The proportional piling exercise was generally well received by experts, who often described it as original and engaging, with some initially perceiving it as game-like. The bean-seed approach appeared visual, intuitive and practical for expressing the relative importance of risk factors and their seasonal variation. It also supported interactive discussion, as participants could review and adjust their allocations during the interview process, although some noted that distributing the seeds across all risk factors required fine-grained comparisons. Future applications of the tool could therefore benefit from a short, structured debriefing to formally document participant experiences, access the acceptability of the method, and identify potential refinements.

A major limitation of this study is the asymmetry in sectoral and disciplinary representation within the expert panel. The use of snowball sampling may have limited the diversity of expertise captured, as participants were identified through existing professional networks. Consequently, some relevant perspectives or areas of disease expertise may have been underrepresented, despite efforts to include experts from different sectors and levels of intervention. For RVF, the focus on animal epidemiology resulted in limited participation from medical professionals. Additionally, the absence of a social science perspective restricted the extend of the analysis, despite the recognised role of human behaviour in zoonotic emergence. A further limitation concerns the predominance of veterinarians in the study sample. While these participants were relevant to the broader One Health scope of the study, their disease-specific expertise may not have been uniform, particularly for EVD. As a result, some perspectives may reflect broader professional experience in zoonoses and surveillance rather than specialised expertise in Ebola itself. This imbalance may be attributed to the voluntary nature of participation, making it challenging to assemble a fully representative panel. In addition, even if grounded in specialised knowledge and field experience, expert knowledge should be interpreted with caution given that Cameroon has yet to document an outbreak of these diseases. The study’s integrated approach averaged expert perceived risk assessments across months, assigning equal weight to all experts. Like previous risk assessment studies based on expert elicitation, expert judgments were aggregated using equal weights, as no empirical calibration data were available to assess relative expert performance in the Cameroonian context. This approach has been applied in comparable settings characterized by high epistemic uncertainty [58]. While this process ensures methodological consistency, it may create explanatory gaps in risk fluctuations. Addressing this through focus group discussions with a more balanced expert panel could refine the contextualization of final analytical outcomes and enhance applicability to disease risk management. Furthermore, integrating complementary quantitative methods, such as longitudinal serological surveys in livestock and wildlife, would strengthen data triangulation and validation, improving the reliability of risk assessments. An important point in interpreting these findings is that neither RVF nor EVD has been officially reported in Cameroon. Expert assessments were therefore based not only on professional experience in surveillance and zoonoses within Cameroon, but also, for several participants, on direct experience managing RVF and Ebola outbreaks in West and Central Africa, including the Senegal and the Democratic Republic of Congo.

An important limitation in interpreting the monthly calendar is the marked ecological heterogeneity of Cameroon. The northern part of the country is characterised by Sahelian and Sudanian ecosystems with a predominantly unimodal rainy season, whereas the southern forested regions follow a more bimodal seasonal pattern. For RVF, temporal patterns are likely to be more influenced by northern livestock-producing and transhumance areas, while for EVD they probably reflect the seasonality of forest-based human-wildlife interfaces in southern and central Cameroon. Because the objective of this study was to generate a national-level anticipatory assessment, expert judgments were intentionally synthesized across regions rather than analysed separately. While this approach provides an overview of perceived emergence risk at the country level, it may obscure ecological and seasonal differences between agro-ecological zones. Regionalized assessments would likely provide more ecologically specific insights and improve the interpretation of temporal risk patterns. As a future direction, it would be valuable to analyse risk dynamics separately across the country’s distinct agro-ecological zones. Such an approach would allow for the assessment of zone-specific emergence patterns, rather than relying solely on a generalized, nationwide evaluation as was done in the present research.

Therefore, the interpretation of temporal weighting for EVD should be made with caution. Unlike RVF, for which seasonal dynamics are relatively well established, the ecology and seasonality of Ebola virus spillover remain incompletely understood, particularly in Cameroon where there is no clear evidence of prior spillover. This uncertainty may have affected the results in two ways. First, the literature-based assessment for EVD was drawn from a more heterogeneous evidence base, in which several factors likely reflected ecological or socio-environmental proxies rather than consistently demonstrated determinants of spillover, as these events are scarce. Second, expert temporal weighting for EVD likely reflected the perceived seasonality of the risk factors identified during the elicitation process, since experts based their monthly weighting on the periods when these risk factors were perceived to be most prominent. The high-risk periods probably reflect the timing of human exposure and human-wildlife interactions more than the actual circulation of the virus or the true timing of spillover events. Therefore, these temporal patterns identified by experts should be considered as indicative and hypothesis-generating. In resources-constrained settings, such indications may nevertheless provide a useful basis for cautiously prioritizing surveillance and prevention activities while additional studies are conducted to refine these findings.

A further limitation of this study is the potential for minor interpretive shifts related to translation. Interviews were conducted and transcribed in French, and the material was later translated into English during manuscript preparation. Although translation was undertaken carefully to preserve the original meaning of participants’ responses, some nuances may have been lost or modified in the process. While this study drew on expertise from national, regional, and local actors, future research could be strengthened by including a broader range of non-official and community-based stakeholders to capture additional experiential knowledge on emergence risk and locally appropriate prevention strategies in Cameroon.

Risk assessments inform decision-making, identify knowledge gaps, and drive research, surveillance, and data collection [59]. Importantly, the identified risk factors are not only relevant to animal and wildlife health, but also to public health preparedness. They point to concrete pathways of human exposure, risk communication needs, and opportunities to prevent human infections at an earlier stage through integrated surveillance as part of the “One Health” approach. In the case of RVF, experts emphasised animal-to-animal transmission in presumed high-risk livestock aggregation and livestock–wildlife interface sites. They also highlighted persistent gaps in the routine documentation of informal, irregular, and cross-border livestock movements, particularly around *Lake Chad* and the *Logone River* basin. While previous work in Cameroon has documented key aspects of the formal cattle trade network and seasonal transhumance [17, 60], our findings indicate that important operational gaps remain for animal traceability, risk-based disease surveillance, and early detection. Two complementary actions could be prioritised, both of which have been implemented in other RVF-endemic settings and shown to be feasible [61, 62]. First, seasonal risk-based surveillance could be strengthened in major livestock congregation areas—including watering points, seasonal grazing areas, stopover camps, and mobile pastoral settlements— in October and November, when herd gatherings peak. Led by veterinary services in close collaboration with public health authorities, local administrations, and laboratory services, this could implement a One Health syndromic surveillance system, monitoring abortions, neonatal mortality, and febrile syndromes in animals, alongside targeted investigation of suspected events. Concurrently, when suspected cases are detected in animals, herd keepers could be briefly surveyed to assess their health status and potential risk practices, such as consuming raw milk or handling sick animals or aborted foetuses, integrating human and animal data for early detection of zoonotic transmission. Second, sentinel surveillance could be piloted in selected urban abattoirs or major destination livestock markets receiving animals from multiple origins, particularly in major urban consumption centres such as Yaoundé and Douala. The need for further risk-mapping studies cannot be overemphasised, as these will be essential to identify high-risk divisions and guide the targeted deployment of surveillance in sentinel herds.

In the case of EVD, experts highlighted the role of high-risk human–wildlife interfaces. Significant operational gaps persist in monitoring these interfaces and in documenting the commercial routes of bushmeat from rural areas to major urban markets, including the species hunted, points of sale, transport nodes, and high-risk handling and consumption practices [63, 64]. To address these gaps, two complementary strategies may be particularly relevant. The first involves establishing sentinel surveillance in selected key bushmeat markets, especially along major trade corridors such as Bertoua–Yaoundé–Douala–Bafoussam, to document species origin, monitor compliance with species protection laws, and, where feasible, perform targeted sampling during periods identified as higher risk by experts (January, May, July, and December). A second strategy could be to strengthen event-based community surveillance to detect unusual wildlife mortality in high-risk divisions and disrupt illegal hunting networks. This approach could include training and equipping wildlife officers, local veterinary health services, ecoguides, and community informants to report unexpected wildlife or NHP deaths, with clear protocols for rapid notification, sample collection, and enforcement. Comparable approaches integrating wildlife mortality monitoring and community event-based surveillance have been applied in other EVD-endemic settings, demonstrating enhanced early outbreak detection. In Gabon and the Republic of Congo, animal mortality networks detected wildlife die-offs prior to human outbreaks, enabling timely response [65], while long-term surveillance models in northern Congo provided operational frameworks for early epizootic detection [66]. In Sierra Leone, community-based event surveillance during the 2015 outbreak improved reporting timeliness and identification of human cases without known epidemiologic links [67]. Finally, further risk-mapping studies remain essential to identify high-risk divisions and to guide the strategic deployment of surveillance in wildlife–human interface areas. The identified environmental, social, and economic determinants, alongside their weightings, form a basis for future research to quantify national risk indices and target areas conducive to pathogen emergence.

## Conclusions

This study emphasizes the importance of spatial determinants in the emergence of RVF in domestic ruminants and the spillover of EVD to humans in Cameroon, drawing on both expert elicitation and scientific literature. It identifies perceived critical periods for the emergence of both diseases, providing actionable evidence to help policymakers target surveillance efforts and optimize resource allocation. The expert-led risk assessment reinforces the bridge between research and policy, supporting improved disease prevention and surveillance across the wildlife value chain, including live wildlife trade and livestock movements. Differences observed between expert-derived and literature-based weightings illustrate the influence of local contextual factors on risk perception and prioritization, highlighting the value of integrating expert insights with literature data to refine risk assessments. In a region consistently identified as at risk for the emergence of these diseases, and in the absence of sufficient epidemiological data for a fully data-driven assessment, the methodology presented here provides a pragmatic foundation for defining intervention levers to guide disease prevention.

## Supplementary Information

Below is the link to the electronic supplementary material.

Supplementary Material 1: Additional file 1- Interview guides.pdf: Semi-structured guides for in-depth individual interviews

Supplementary Material 2: Additional file 2- Experience characterization grid.pdf: Grid with sections for characterising the experience of each stakeholder

Supplementary Material 3: Additional file 3- Proportional piling.pdf: Images of the scoring process using the monthly calendar

Supplementary Material 4: Additional file 4- Weights from the literature.pdf: Tables summarising the number of occurrences in scientific literature for each risk factor and determining their relative weights

Supplementary Material 5: Additional file 5- Distribution of participants across disease-specific interview modules.xlsx: Breakdown of the 22 unique experts by interview module (EVD only, RVF only, and both)

Supplementary Material 6: Additional file 6- Risk factors definitions.pdf: Definition of all the terms used to describe each risk factor

Supplementary Material 7: Additional file 7- Box plots illustrating the variability in expert weightings RVF.pdf: For each month, the variability of the risk scores assigned by experts to RVF key risk factors was represented by box plots

Supplementary Material 8: Additional file 8- Box plots illustrating the variability in expert weightings EVD.pdf: For each month, the variability of the risk scores assigned by experts to EVD key risk factors was represented by box plots

Supplementary Material 9: Additional file 9- Individual relative discrepancies RVF.pdf: Discrepancies between experts and scientific literature regarding the importance of each RVF risk factor

Supplementary Material 10: Additional file 10- Individual relative discrepancies EVD.pdf: Discrepancies between experts and scientific literature regarding the importance of each EVD risk factor

## Data Availability

The data from which the results have been obtained in this manuscript will be available on request from the authors, according to compliance with the General Data Protection Regulation (GDPR).

## References

[1] Koua EL, Njingang JRN, Kimenyi JP, Williams GS, Okeibunor J, Oka S, et al. Trends in public health emergencies in the WHO African Region: an analysis of the past two decades public health events from 2001 to 2022. BMJ Glob Health. 2023;8(10):e012015. 10.1136/bmjgh-2023-012015. PubMed PMID: 37813470; PubMed Central PMCID: PMC10565238. PMC10565238 37813470

[2] Poungou N, Sevidzem SL, Koumba AA, Koumba CRZ, Mbehang P, Onanga R, et al. Mosquito-Borne Arboviruses Occurrence and Distribution in the Last Three Decades in Central Africa: A Systematic Literature Review. Microorganisms. 2024;12(1):4. 10.3390/microorganisms12010004. PMC10819313 38276174

[3] Rugarabamu S, Mwanyika GO, Rumisha SF, Sindato C, Lim HeeYoung, Misinzo G, et al. Seroprevalence and associated risk factors of selected zoonotic viral hemorrhagic fevers in Tanzania. Int J Infect Dis. 2021;109:174–81. 10.1016/j.ijid.2021.07.006. Located at: lah. 34242761

[4] Nyakarahuka L, Kankya C, Krontveit R, Mayer B, Mwiine FN, Lutwama J, et al. How severe and prevalent are Ebola and Marburg viruses? A systematic review and meta-analysis of the case fatality rates and seroprevalence. BMC Infect Dis. 2016;16:708. 10.1186/s12879-016-2045-6. PubMed PMID: 27887599; PubMed Central PMCID: PMC5124280. PMC5124280 27887599

[5] Gonzalez JP, Souris M, Valdivia-Granda W. Global spread of hemorrhagic fever viruses: Predicting pandemics. Methods Mol Biol. 2018;1604:3–31. 10.1007/978-1-4939-6981-4_1. Located at: Scopus. PMC7120037 28986822

[6] Olivero J, Fa JE, Real R, Farfán MÁ, Márquez AL, Vargas JM, et al. Mammalian biogeography and the Ebola virus in Africa. Mammal Rev. 2017;47(1):24–37. 10.1111/mam.12074.

[7] Rissmann M, Eiden M, Wade A, Poueme R, Abdoulkadiri S, Unger H, et al. Evidence for enzootic circulation of Rift Valley fever virus among livestock in Cameroon. Acta Trop. 2017;172:7–13. 10.1016/j.actatropica.2017.04.001. 28427961

[8] Mpouam SE, Ikoum D, Hadja L, Mingoas JPK, Saegerman C. Parallel multi-criteria decision analysis for sub-national prioritization of zoonoses and animal diseases in Africa: The case of Cameroon. PLoS ONE. 2024;19(6):e0295742. 10.1371/journal.pone.0295742. PMC11198839 38917073

[9] CDC. Zoonotic disease prioritization for inter-sectoral engagement in Cameroon. 2016.

[10] MINEPIA. Directives de la surveillance en santé animale dans la communauté: Cas des zoonoses prioritaires. 2021.

[11] Chowell G, Nishiura H. Transmission dynamics and control of Ebola virus disease (EVD): a review. BMC Med. 2014;12(1):196. 10.1186/s12916-014-0196-0. PMC4207625 25300956

[12] Sopbué Kamguem I, Kirschvink N, Wade A, Linard C. Determinants of viral haemorrhagic fever risk in Africa’s tropical moist forests: A scoping review of spatial, socio-economic, and environmental factors. PLoS Negl Trop Dis. 2025;19(1):e0012817. 10.1371/journal.pntd.0012817. 39820141

[13] Obeng-Kusi M, Martin J, Abraham I. The economic burden of Ebola virus disease: a review and recommendations for analysis. J Med Econ. 2024;27(1):309–23. 10.1080/13696998.2024.2313358. PubMed PMID: 38299454. 38299454

[14] Sado FY, Tchetgna HS, Kamgang B, Djonabaye D, Nakouné E, McCall PJ, et al. Seroprevalence of Rift Valley fever virus in domestic ruminants of various origins in two markets of Yaoundé, Cameroon. PLoS Negl Trop Dis. 2022;16(8):e0010683. 10.1371/journal.pntd.0010683. PMC9397978 35951644

[15] Kossebe CF, Tchobsala, Danra Djackba D, Baye-Niwah C. Endogenous knowledge and structures of parks dominated by Adansonia digitata (Baobab) species in the Sudano-Sahelian agro-ecological zone of Cameroon. Environ Chall. 2022;9:100622. 10.1016/j.envc.2022.100622.

[16] Vondou DA, Guenang GM, Djiotang TLA, Kamsu-Tamo PH. Trends and Interannual Variability of Extreme Rainfall Indices over Cameroon. Sustainability. 2021;13(12):6803. 10.3390/su13126803.

[17] Motta P, Porphyre T, Handel IG, Hamman SM, Ngu Ngwa V, Tanya VN, et al. Characterizing livestock markets, primary diseases, and key management practices along the livestock supply chain in Cameroon. Front Vet Sci. 2019;6. 10.3389/fvets.2019.00101. PMC6467964 31024939

[18] Tsapi V, Assene MN, Haasis HD. The Complexity of the Meat Supply Chain in Cameroon: Multiplicity of Actors, Interactions and Challenges. Logistics. 2022;6(4):4. 10.3390/logistics6040086.

[19] Institut National de la Statistique du Cameroun. Annuaire, statistique du sous-secteur de l’elevage, des peches et, des industries animales (Edition. 2021) [Internet]. 2023 [cited 2025 May 4]. Available from: https://ins-cameroun.cm/document/annuaire-statistique-du-sous-secteur-de-lelevage-des-peches-et-des-industries-animales-edition-2021/.

[20] Pomeroy LW, Moritz M, Garabed R. Network analyses of transhumance movements and simulations of foot-and-mouth disease virus transmission among mobile livestock in Cameroon. Epidemics. 2019;28:100334. 10.1016/j.epidem.2019.02.005. 31387783

[21] Ebogo-Belobo JT, Sadeuh-Mba SA, Chavely GM, Groschup MH, Mbacham WF, Njouom R. First detection of Rift Valley fever virus antibodies in non-human primates in Cameroon. J Med Primatol. 2024;53(1):e12687. 10.1111/jmp.12687. PubMed PMID: 38083993. 38083993

[22] Poueme R, Stoek F, Nloga N, Awah-Ndukum J, Rissmann M, Schulz A, et al. Seroprevalence and Associated Risk Factors of Rift Valley Fever in Domestic Small Ruminants in the North Region of Cameroon. Vet Med Int. 2019;2019:e8149897. 10.1155/2019/8149897. PMC6925726 31885848

[23] Judson SD, LeBreton M, Fuller T, Hoffman RM, Njabo K, Brewer TF, et al. Translating Predictions of Zoonotic Viruses for Policymakers. EcoHealth. 2018;15(1):52–62. 10.1007/s10393-017-1304-3. 29230614

[24] Colby LA, Zitzow L. Applied Institutional Approaches for the Evaluation and Management of Zoonoses in Contemporary Laboratory Animal Research Facilities. ILAR J. 2018;59(2):134–43. 10.1093/ilar/ily. 016 PubMed PMID: 30541146. 30541146

[25] Tsige TZ, Shapa Y, Endale Z. One health approach for prevention and control of zoonotic diseases. Rev. 2020;99–106. 10.5829/idosi.gv.2020.99.106.

[26] Gaddy HG. Using local knowledge in emerging infectious disease research. Soc Sci Med. 2020;258:113107. 10.1016/j.socscimed.2020.113107. PMC7292947 32563166

[27] Kelly TR, Karesh WB, Johnson CK, Gilardi KVK, Anthony SJ, Goldstein T, et al. One Health proof of concept: Bringing a transdisciplinary approach to surveillance for zoonotic viruses at the human-wild animal interface. Prev Vet Med. 2017; Special Issue on 14th International Symposium of Veterinary Epidemiology and Economics, Yucatan, Mexico 2015;137:112–8. 10.1016/j.prevetmed.2016.11.023. PMC7132382 28034593

[28] Morse SS, Mazet JA, Woolhouse M, Parrish CR, Carroll D, Karesh WB, et al. Prediction and prevention of the next pandemic zoonosis. Lancet. 2012;380(9857):1956–65. 10.1016/S0140-6736(. 12)61684-5 PubMed PMID: 23200504. PMC3712877 23200504

[29] Alders RG, Ali SN, Ameri AA, Bagnol B, Cooper TL, Gozali A, et al. Participatory Epidemiology: Principles, Practice, Utility, and Lessons Learnt. Front Vet Sci. 2020;7:532763. 10.3389/fvets.2020.532763. PubMed PMID: 33330678; PubMed Central PMCID: PMC7672004. PMC7672004 33330678

[30] Squarzoni-Diaw C, Arsevska E, Kalthoum S, Hammami P, Cherni J, Daoudi A, et al. Using a participatory qualitative risk assessment to estimate the risk of introduction and spread of transboundary animal diseases in scarce-data environments. Transbound Emerg Dis. 2021;68(4):1966–78. 10.1111/tbed.13920. 33174371

[31] Jost CC, Mariner JC, Roeder PL, Sawitri E, Macgregor-Skinner GJ. Participatory epidemiology in disease surveillance and research. 2007;3(26):537–47. 18293603

[32] Wirsiy FS, Boock AU, Akoachere JFTK. Assessing the determinants of Ebola virus disease transmission in Baka Community of the Tropical Rainforest of Cameroon. BMC Infect Dis. 2021;21(1):324. 10.1186/s12879-021-06011-z. PubMed PMID: 33827424; PubMed Central PMCID: PMC8028822. PMC8028822 33827424

[33] Thrusfield M, Christley R. Veterinary epidemiology, 4th edition. Wiley-Blackwell. 2018. 896 p.

[34] Malczewski J. GIS and multicriteria decision analysis. Wiley; 1999. p. 408.

[35] Catley A, Osman J, Mawien C, Jones BA, Leyland TJ. Participatory analysis of seasonal incidences of diseases of cattle, disease vectors and rainfall in southern Sudan. Prev Vet Med. 2002;53(4):275–84. 10.1016/S0167-5877(01)00289-6. 11937234

[36] Stevens KB, Gilbert M, Pfeiffer DU. Modeling habitat suitability for occurrence of highly pathogenic avian influenza virus H5N1 in domestic poultry in Asia: A spatial multicriteria decision analysis approach. Spat Spatio-Temporal Epidemiol. 2013;4:1–14. 10.1016/j.sste.2012.11.002. 23481249

[37] van Doorn HR. Emerging infectious diseases. Medicine (Baltimore). 2014; Infections part 3 of 342(1):60–3. 10.1016/j.mpmed.2013.10.014. PMC3929004 24563608

[38] Catley A, Alders RG, Wood JLN. Participatory epidemiology: approaches, methods, experiences 1997. Vet J Lond Engl. 2012;191(2):151–60. 10.1016/j.tvjl.2011.03.010. PubMed PMID: 21856195. 21856195

[39] Malterud K, Siersma VD, Guassora AD. Sample Size in Qualitative Interview Studies: Guided by Information Power. Qual Health Res. 2016;26(13):1753–60. 10.1177/1049732315617444 PubMed PMID: 26613970. 26613970

[40] Guest G, Bunce A, Johnson L. How Many Interviews Are Enough? An Experiment with Data Saturation and Variability. Field Methods. 2006;18(1):59–82. 10.1177/1525822X05279903.

[41] Battistella L, et al. Terrestrial ecoregions of the world: a new map of life on Earth. Bioscience. 2001;51(11):933–8.

[42] Schmid P, Rauber D, Betsch C, Lidolt G, Denker ML. Barriers of Influenza Vaccination Intention and Behavior – A Systematic Review of Influenza Vaccine Hesitancy, 2005–2016. PLoS ONE. 2017;12(1):e0170550. 10.1371/journal.pone.0170550. PMC5268454 28125629

[43] Sarikhani Y, Ghahramani S, Bayati M, Lotfi F, Bastani P. A thematic network for factors affecting the choice of specialty education by medical students: a scoping study in low-and middle-income countries. BMC Med Educ. 2021;21:99. 10.1186/s12909-021-02539-5. PubMed PMID: 33568113; PubMed Central PMCID: PMC7877062. PMC7877062 33568113

[44] Giavarina D. Understanding Bland Altman analysis. Biochem Med. 2015;25(2):141–51. 10.11613/BM.2015.015. PMC4470095 26110027

[45] Bland JM, Altman DG. Statistical methods for assessing agreement between two methods of clinical measurement. Lancet. 1986;1(8476):307–10. PubMed PMID: 2868172. 2868172

[46] R Core Team. R: A language and environment for statistical computing. R Foundation for Statistical Computing. Vienna: Vienna, Austria: R Foundation for Statistical Computing, Vienna, Austria;; 2022. Austria. URL. https://www.R-project.org/.

[47] CNIL. L’anonymisation des données, un traitement clé pour l’open data [Internet]. 2019 [cited 2024 Apr 11]. Available from: https://www.cnil.fr/fr/lanonymisation-des-donnees-un-traitement-cle-pour-lopen-data.

[48] Krippendorff K. Content analysis: an introduction to its methodology. 3rd ed. SAGE; 2013. 457 p.

[49] Kainga H, Phonera MC, Chatanga E, Kallu SA, Mpundu P, Samutela M, et al. Seroprevalence and Associated Risk Factors of Rift Valley Fever in Livestock from Three Ecological Zones of Malawi. Pathogens. 2022;11(11):11. 10.3390/pathogens11111349. PMC9698272 36422600

[50] Abdallah MMM, Adam IA, Abdalla TM, Abdelaziz SA, Ahmed ME, Aradaib IE. A survey of rift valley fever and associated risk factors among the one-humped camel (Camelus dromedaries) in Sudan. Ir Vet J. 2016;69(1):6. 10.1186/s13620-016-0065-6. PMC4901444 27293548

[51] Lancelot R, Béral M, Rakotoharinome VM, Andriamandimby SF, Héraud JM, Coste C, et al. Drivers of Rift Valley fever epidemics in Madagascar. Proc Natl Acad Sci. 2017;114(5):938–43. 10.1073/pnas.1607948114. PMC5293023 28096420

[52] Sang R, Lutomiah J, Said M, Makio A, Koka H, Koskei E, et al. Effects of Irrigation and Rainfall on the Population Dynamics of Rift Valley Fever and Other Arbovirus Mosquito Vectors in the Epidemic-Prone Tana River County, Kenya. J Med Entomol. 2017;54(2):460–70. 10.1093/jme/tjw206. PMC5850818 28011732

[53] Evans TS, Tutaryebwa L, Gilardi KV, Barry PA, Marzi A, Eberhardt M, et al. Suspected exposure to filoviruses among people contacting wildlife in Southwestern Uganda. J Infect Dis. 2018; Special issue: Marburg and Ebola viruses: marking 50 years since discovery. 218(Suppl. 5):S277–86. Located at: lah. 10.1093/infdis/jiy251. PMC6927880 29924324

[54] Paige SB, Frost SDW, Gibson MA, Holland J, Shankar A, Switzer WM, et al. Beyond bushmeat: Animal contact, injury, and zoonotic disease risk in western Uganda. EcoHealth. 2014;11(4):534–43. 10.1007/s10393-014-0942-y. PubMed PMID: 24845574; PubMed Central PMCID: PMC4240769. PMC4240769 24845574

[55] Hitziger M, Antoine-Moussiaux N, Morales DR, Wernli D. Knowledge integration in one health: science and decision support. In: principles of one health for a better planet [Internet]. 2025 [cited 2025 Apr 25]. p. 73–92. (CABI Books). Available from: https://www.cabidigitallibrary.org/doi/abs/10.1079/9781800623002.0005.

[56] Alles M, Eussen S, Ake-Tano O, Diouf S, Tanya A, Lakati A, et al. Situational analysis and expert evaluation of the nutrition and health status of infants and young children in five countries in sub-Saharan Africa. Food Nutr Bull. 2013;34(3):287–98. 10.1177/156482651303400301. PubMed PMID: 24167909. 24167909

[57] Lane-Fall MB. Why Epidemiology Is Incomplete Without Qualitative and Mixed Methods. Am J Epidemiol. 2023;192(6):853–5. 10.1093/aje/kwad050. 36890115

[58] Sebok E, Henriksen HJ, Pastén-Zapata E, Berg P, Thirel G, Lemoine A, et al. Use of expert elicitation to assign weights to climate and hydrological models in climate impact studies. Hydrol Earth Syst Sci. 2022;26(21):5605–25. 10.5194/hess-26-5605-2022.

[59] Grace D, Monda J, Karanja N, Randolph TF, Kang’ethe EK. Participatory probabilistic assessment of the risk to human health associated with cryptosporidiosis from urban dairying in Dagoretti, Nairobi, Kenya. Trop Anim Health Prod. 2012;44(S1):33–40. 10.1007/s11250-012-0204-3. 22886443

[60] Motta P, Porphyre T, Handel I, Hamman SM, Ngu Ngwa V, Tanya V, et al. Implications of the cattle trade network in Cameroon for regional disease prevention and control. Sci Rep. 2017;7(1):43932. 10.1038/srep43932. PMC5339720 28266589

[61] Oyas H, Holmstrom L, Kemunto NP, Muturi M, Mwatondo A, Osoro E, et al. Enhanced surveillance for Rift Valley Fever in livestock during El Niño rains and threat of RVF outbreak, Kenya, 2015–2016. PLoS Negl Trop Dis. 2018;12(4):e0006353. 10.1371/journal.pntd.0006353. PMC5919633 29698487

[62] Gerken KN, Ndenga BA, Owuor KO, Winter CA, Seetah K, LaBeaud AD. Leveraging livestock movements to urban slaughterhouses for wide-spread Rift Valley fever virus surveillance in Western Kenya. One Health. 2022;15:100457. 10.1016/j.onehlt.2022.100457. PMC9754961 36532672

[63] TRAFFIC. Développement d’un système de suivi de la viande de brousse en Afrique Centrale (SYVBAC) [Internet]. 2022 [cited 2026 Mar 14]. Available from: https://www.traffic.org/publications/reports/development-of-a-monitoring-system-for-bushmeat-in-central-africa-fr/.

[64] TRAFFIC. Risk analysis of zoonosis along the bushmeat value chain in Cameroon - wildlife trade report from TRAFFIC [Internet]. 2024 [cited 2026 Mar 14]. Available from: https://www.traffic.org/publications/reports/risk-analysis-of-zoonosis-along-the-bushmeat-value-chain-in-cameroon/.

[65] Rouquet P, Froment JM, Bermejo M, Kilbourn A, Karesh W, Reed P, et al. Wild animal mortality monitoring and human Ebola outbreaks, Gabon and Republic of Congo, 2001–2003. Emerg Infect Dis. 2005;11(2):283–90. 10.3201/eid1102. 040533 PubMed PMID: 15752448; PubMed Central PMCID: PMC3320460. PMC3320460 15752448

[66] Kuisma E, Olson SH, Cameron KN, Reed PE, Karesh WB, Ondzie AI, et al. Long-term wildlife mortality surveillance in Northern Congo: a model for the detection of Ebola virus disease epizootics. Philos Trans R Soc B Biol Sci. 2019;374(1782). Located at: lah. 10.1098/rstb.2018.0339. PMC6711308 31401969

[67] Ratnayake R, Crowe SJ, Jasperse J, Privette G, Stone E, Miller L, et al. Assessment of Community Event-Based Surveillance for Ebola Virus Disease, Sierra Leone, 2015. Emerg Infect Dis. 2016;22(8):1431–7. 10.3201/eid2208. 160205 PubMed PMID: 27434608; PubMed Central PMCID: PMC4982166. PMC4982166 27434608

