# Supplementary material for: A participatory expert-based risk assessment to inform zoonotic disease surveillance in Cameroon: the case of Ebola virus disease and Rift Valley fever
Source: One Health Outlook. 2026 Aug 7;8:55. doi: 10.1186/s42522-026-00232-5 (PMC13732831; doi:10.1186/s42522-026-00232-5)
Supplement: Supplementary file 1 [file 42522_2026_232_MOESM10_ESM.pdf]

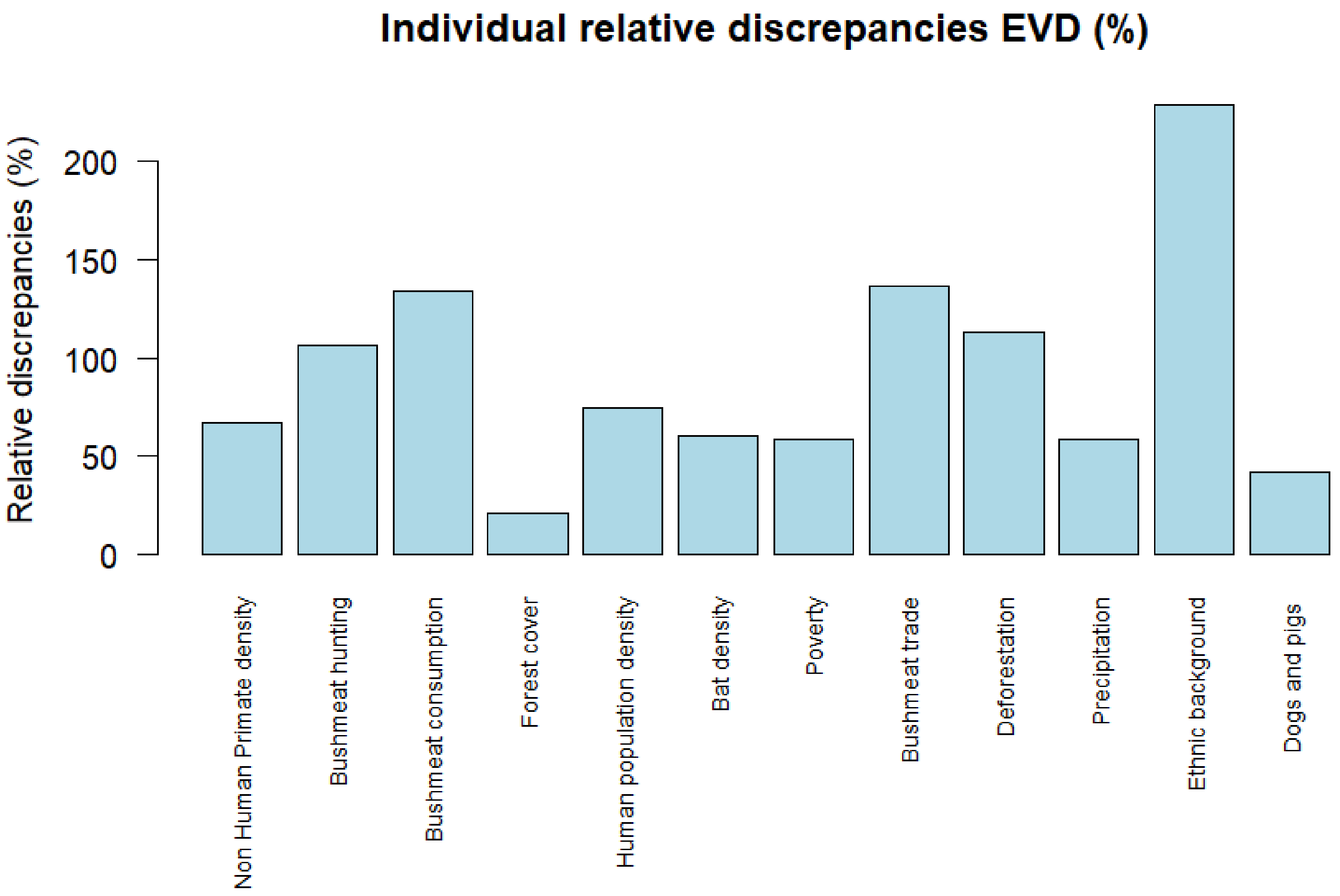
Individual relative discrepancies EVD (%)
Relative discrepancies (%)
0
50
100
150
200
Non Human Primate density
Bushmeat hunting
Bushmeat consumption
Forest cover
Human population density
Bat density
Poverty
Bushmeat trade
Deforestation
Precipitation
Ethnic background
Dogs and pigs
