# Supplementary material for: A participatory expert-based risk assessment to inform zoonotic disease surveillance in Cameroon: the case of Ebola virus disease and Rift Valley fever
Source: One Health Outlook. 2026 Aug 7;8:55. doi: 10.1186/s42522-026-00232-5 (PMC13732831; doi:10.1186/s42522-026-00232-5)
Supplement: Supplementary file 2 [file 42522_2026_232_MOESM1_ESM.pdf]

INTERVIEW GUIDES

A. Interview guide for Rift Valley Fever

- In Cameroon, what do you think are the months when the risk of transmission from an animal to another is highest? (Proportional piling)

- What factors do you think would contribute to an outbreak and spread of RVF within the different animal host populations (cattle, camelids, small ruminants)? (In other words, we could also ask: What are the spatial factors that make village X more at risk of harbouring an animal RVF outbreak than village Y?)

- (Based on an exhaustive list of risk factors presented[1]): What do you think of these different spatial risk factors identified in the literature?

- Based on the above, would you like to modify the list of factors you have previously drawn up? If so, how would you modify it?

- For each month of the year, estimate for each factor considered its importance (or its contribution) in relation to the other factors, to the transmission of RVF from one infected animal to another (proportional piling).

[1] List of spatial risk factors identified in the literature

Rift Valley Fever
Transmission within the animal population
Climate:
- Heavy rain and flooding
- Average temperature [24.2-25.5°C]
- El Nino Southern Oscillation (ENSO)
Land cover:
- Vegetation index (Bushy vegetation)
- Rivers and streams
- Water bodies (lakes, stagnant water, ponds, marshes, flood plains, etc.)
- Forest
Land use:
- Deforestation
- Irrigation
- Rainfed crops
- Fish ponds

**Topography:**
- Low altitude
**Vector/animal host/reservoir:**
- Mosquito density and diversity
- High density of ruminants (cattle, camelids, small ruminants, especially sheep)
- Various domestic vertebrate hosts (including goats, dogs, cats, horses, donkeys and poultry)
- Wildlife (African buffalo, giraffes, desert warthogs, elephants, rhinoceroses, antelopes, other wild ruminants)
- Transhumance and nomadism
- Trade in ruminants
**Anthropogenic factors:**
- Water storage methods (e.g. conserving water in small dams)
- Deforestation
- Urbanisation (urban areas)
- Conflict and political instability

B. Interview guide for Ebola Virus Disease

- In Cameroon, what do you think are the months when the risk of transmission from animals to humans is highest? (Proportional piling)

- In Cameroon, what do you think are all the factors that would encourage humans to be contaminated by an animal infected with the Ebola virus? (In other words, we could also ask: What are the spatial factors that make village X more at risk of zoonotic transmission of the Ebola virus than another village Y?)

- (Based on an exhaustive list of risk factors presented[2]): What do you think of these different spatial risk factors identified in the literature? In what way do you think they reflect the reality on the ground?

- In view of the above, would you like to modify the list of factors you have drawn up? If so, how?

- For each month of the year, estimate for each factor considered its importance (or its contribution) in relation to the other factors, to the passage of the Ebola virus from animals to humans (proportional piling).

[2]List of spatial risk factors identified in the literature

Ebola Virus Disease
Spillover
Climate:<br>- Average monthly precipitation<br>- Low annual temperature range<br>- Soil surface temperature<br>- Evapotranspiration<br>Land cover/use:<br>- Forest<br>- Cultivated land<br>Topography:<br>- Altitude<br>Vector/animal host/reservoir:<br>- Rodents (bush rats, porcupines, etc.)<br>- Bats<br>- duikers (antelopes)<br>- Non-human primates (monkeys, chimpanzees, gorillas)<br>- Wild Suids (bush pigs, wild boars, etc.)<br>- Dogs and pigs<br>Anthropogenic factors:<br>- Human population density<br>- Socio-cultural practices linked to livelihood and forest-based subsistence<br>- Poverty (per capita income, per capita gross domestic product, housing conditions)<br>- Level of education<br>- Deforestation<br>- Hunting, trade and consumption of wild animals<br>- Conflict and political instability
