# Supplementary material for: A participatory expert-based risk assessment to inform zoonotic disease surveillance in Cameroon: the case of Ebola virus disease and Rift Valley fever
Source: One Health Outlook. 2026 Aug 7;8:55. doi: 10.1186/s42522-026-00232-5 (PMC13732831; doi:10.1186/s42522-026-00232-5)
Supplement: Supplementary file 3 [file 42522_2026_232_MOESM2_ESM.pdf]

EXPERT PROFILE AND DISEASE-SPECIFIC EXPERIENCE

I- GENERAL PROFILE OF THE EXPERT

1- What is your profession (Multiple answers possible):

- ☐ Medical doctor
- ☐ Medical nurse
- ☐ Medical laboratory technician
- ☐ Veterinary doctor
- ☐ Veterinary nurse
- ☐ Veterinary laboratory technician
- ☐ Zootechnician
- ☐ Agricultural engineer
- ☐ Wildlife officer
- ☐ Animal health authority/Policy maker
- ☐ Public health authority/Policy maker
- ☐ Researcher
- ☐ Lecturer/ Teacher
- ☐ Other (Please specify) ....

2- What is your sector of employment? (Multiple answers possible):

- ☐ Public
- ☐ Private
- ☐ Both
- ☐ Other (Please specify) ....

3- What is your level of intervention in the health pyramid (Multiple answers possible):

- ☐ National
- ☐ Regional
- ☐ Divisional level
- ☐ Health District
- ☐ Other (Please specify) ....

4- In which sectors do you work? (Multiple answers possible)

- ☐ Livestock
- ☐ Agriculture
- ☐ Public health
- ☐ Wildlife
- ☐ Forests
- ☐ Environmental
- ☐ Public administration- Political decision-maker
- ☐ Other (Please specify) …………………………………………………..

5- Please indicate your areas of professional practice:

Country(ies): ……

Region(s): ………

Division(s): ……

II- DISEASE SPECIFIC KNOWLEDGE AND EXPERIENCE

A/ Rift Valley Fever

On a scale of 1-10, how would you rate yourself in the following areas?

1- Knowledge of the biology (pathogenesis/infectivity) of the pathogen /10

2- Knowledge of the mechanisms by which pathogens are transmitted to hosts /10

3- Clinical knowledge of the disease in animals /10

4- Clinical knowledge of the disease in humans /10

5- Have you ever been professionally exposed to a suspected or confirmed infected animal or human case?

- ☐ Yes
- ☐ No

6- How often are you exposed to information, activities, or professional tasks related to this disease?

- ☐ Very frequently (almost every month)
- ☐ Frequently (at least twice a year)
- ☐ Rarely (at least once a year)
- ☐ Very rare (at least once during working career)
- ☐ Never
- ☐ Other (Please specify) ………………………………………………………..

7- How long have you been involved in activities related to this disease?

- ☐ < 5 years
- ☐ 5-10 years
- ☐ 10-15 years
- ☐ >15 years

B/ Ebola Virus Disease

On a scale of 1-10, how would you rate yourself in the following areas?

1- Knowledge of the biology (pathogenesis/infectivity) of the pathogen /10

2- Knowledge of the mechanisms by which pathogens are transmitted to hosts /10

3- Clinical knowledge of the disease in animals /10

4- Clinical knowledge of the disease in humans /10

5- Have you ever been professionally exposed to a suspected or confirmed infected animal or human case?

- ☐ Yes
- ☐ No

6- How often are you exposed to information, activities, or professional tasks related to this disease?

- ☐ Very frequently (almost every month)
- ☐ Frequently (at least twice a year)
- ☐ Rarely (at least once a year)
- ☐ Very rare (at least once during working career)
- ☐ Never
- ☐ Other (Please specify) ……………………………………………………..

7- How long have you been involved in activities related to this disease?

- ☐ < 5 years
- ☐ 5-10 years
- ☐ 10-15 years
- ☐ >15 years
