# Supplementary material for: A participatory expert-based risk assessment to inform zoonotic disease surveillance in Cameroon: the case of Ebola virus disease and Rift Valley fever
Source: One Health Outlook. 2026 Aug 7;8:55. doi: 10.1186/s42522-026-00232-5 (PMC13732831; doi:10.1186/s42522-026-00232-5)
Supplement: Supplementary file 4 [file 42522_2026_232_MOESM3_ESM.pdf]

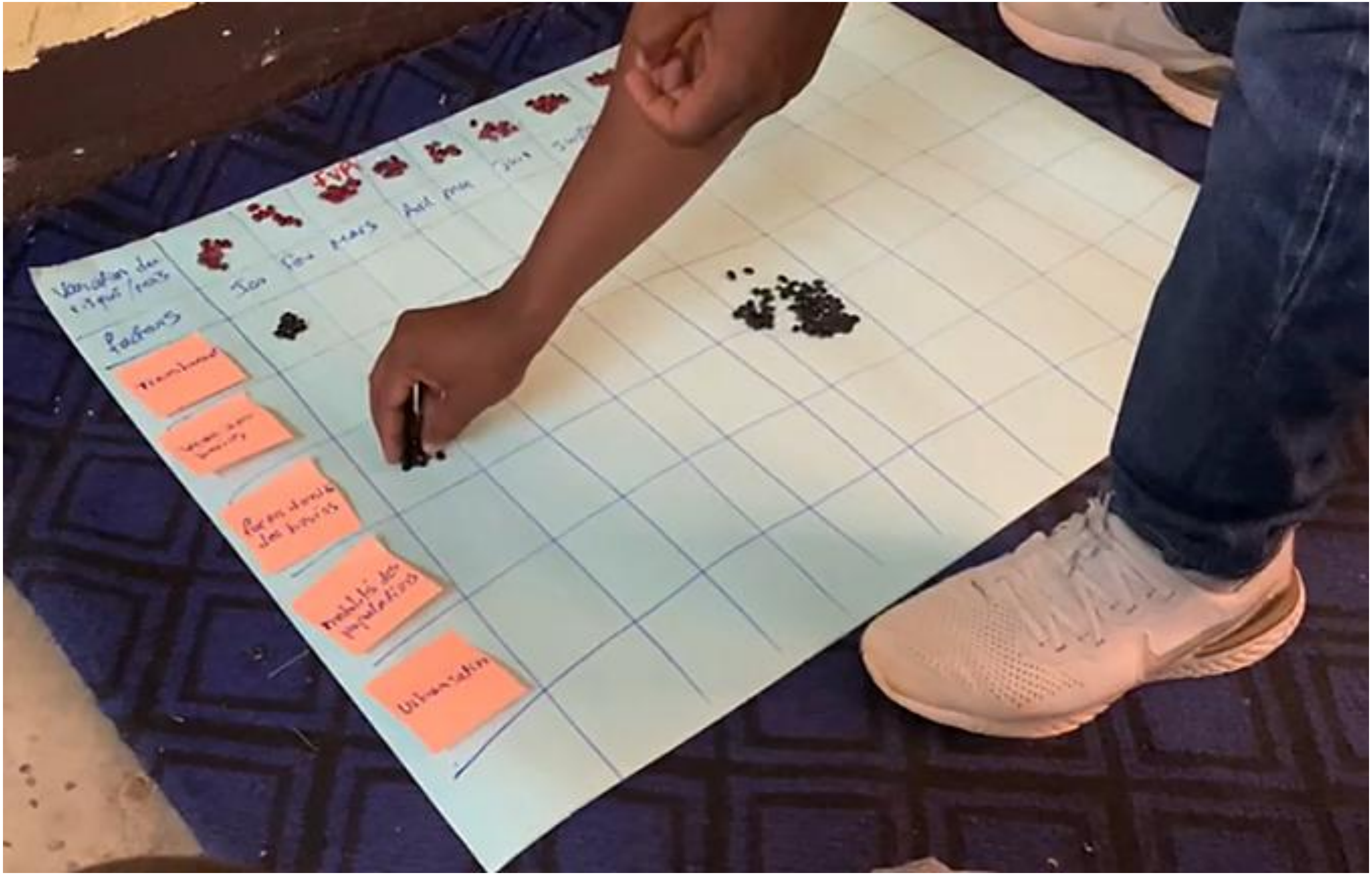
Facteurs
mars
Urbanisation

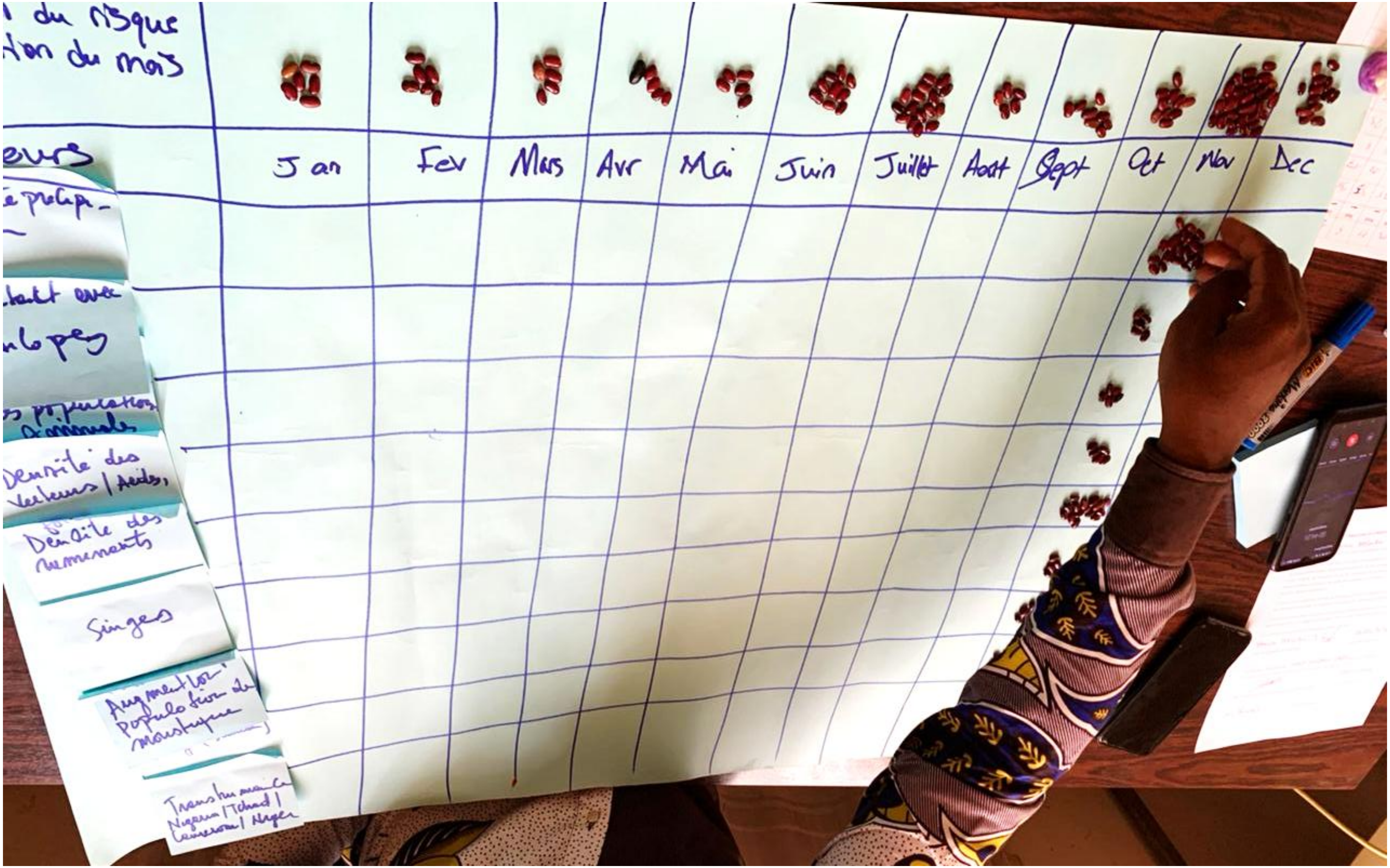
du risque
tion du mois
Jan
Fev
Mars
Avr
Mai
Juin
Juillet
Aout
Sept
Oct
Nov
Dec
Densité des vecteurs / Aedes,
Singes
Transhumance
Nigeria / Tchad /
Cameroun / Niger

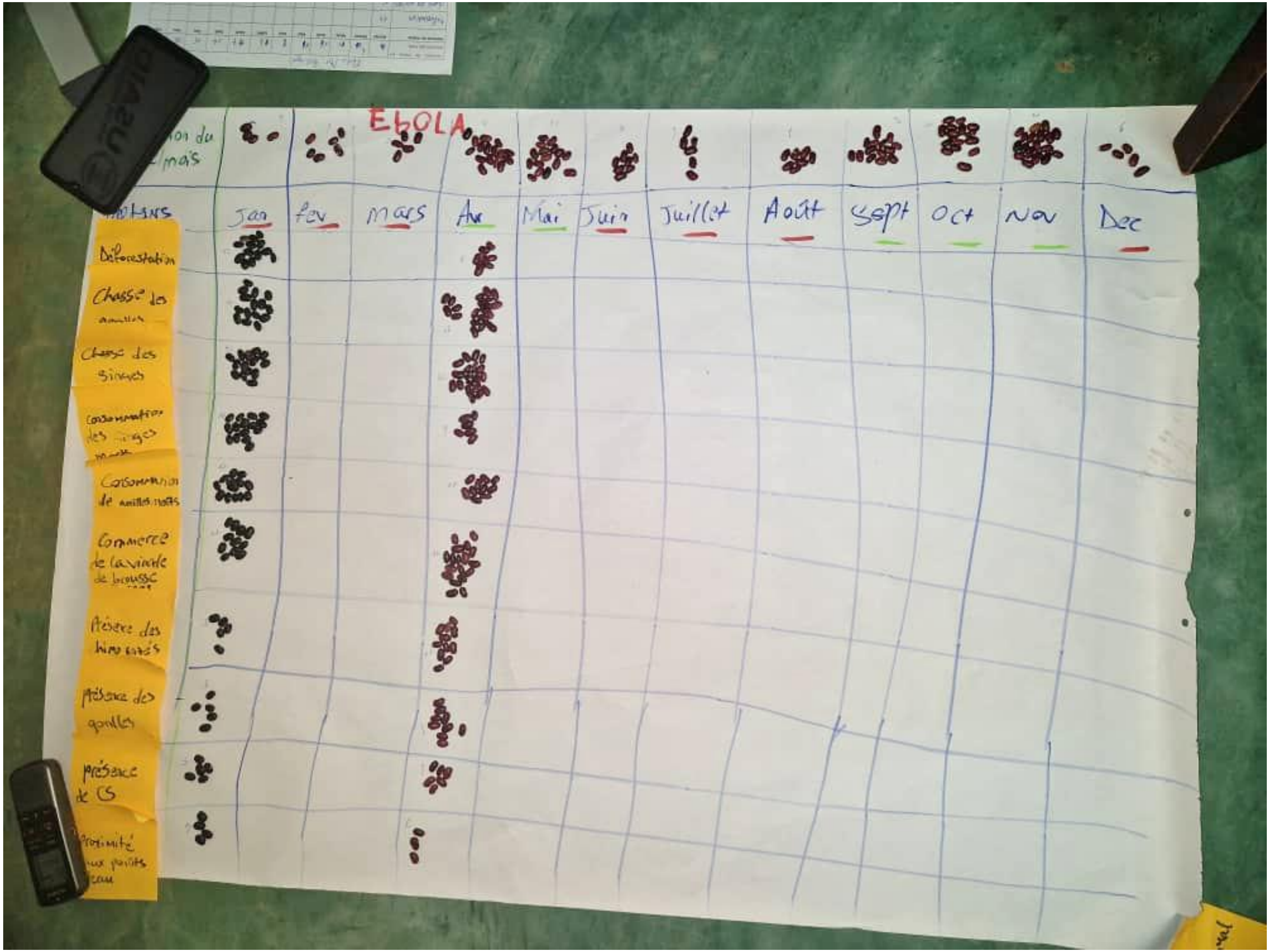
EBOLA
Jan
fev
mars
Avr
Mai
Juin
Juillet
Août
Sept
Oct
Nov
Dec
Déforestation
Chasse des
Chasse des singes
Consommation de animaux morts
Commerce de la viande de brousse
Présence des
présence des gorilles
présence de CS
Proximité aux points

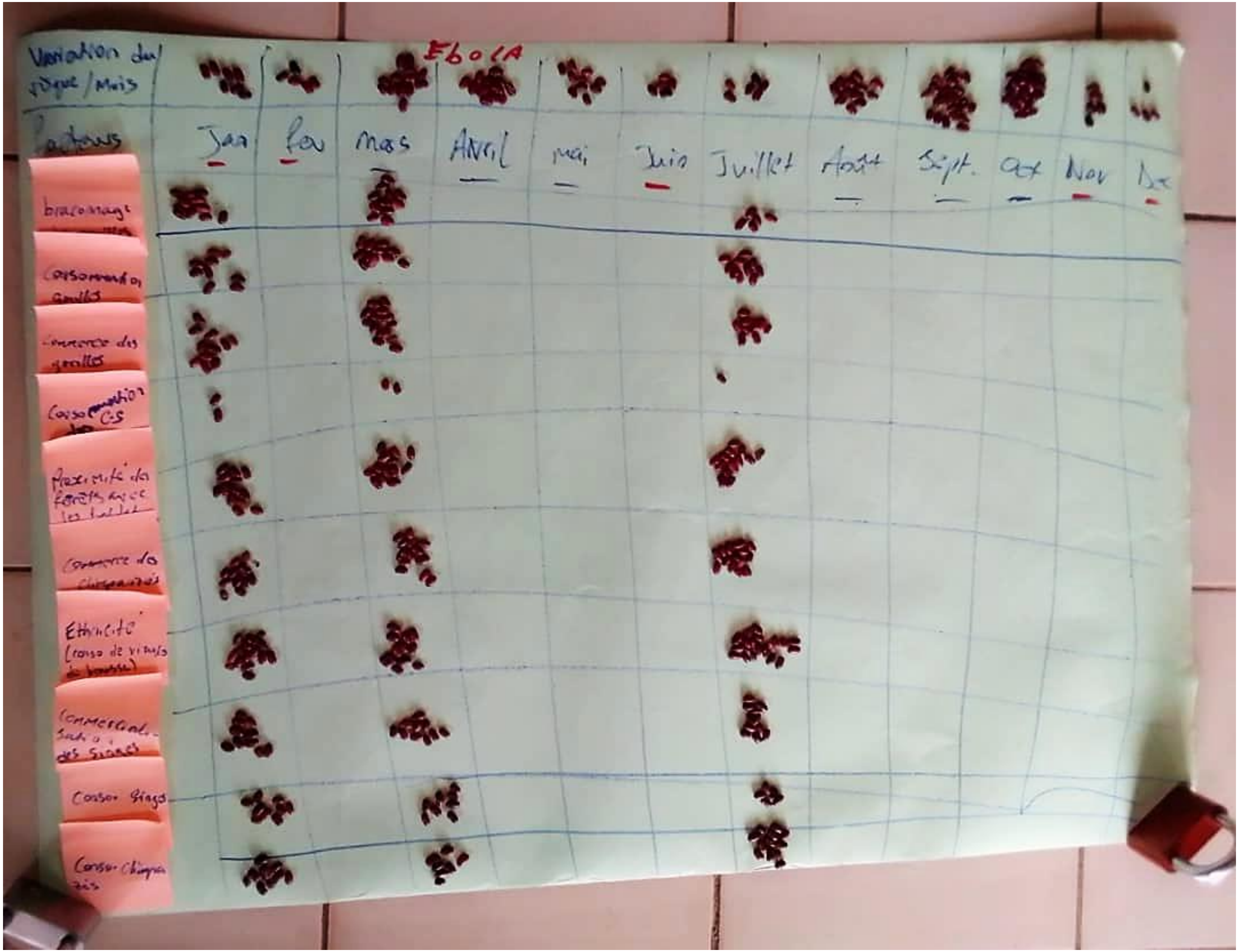
Ebola
Jan
Mars
Avril
mai
Juin
Juillet
Sept.
Oct
Nov
braconnage
Commerce des gorilles
Consommation C.S
Commerce des chimpanzés
Ethnicité (conso de viande de brousse)
Conso. singes

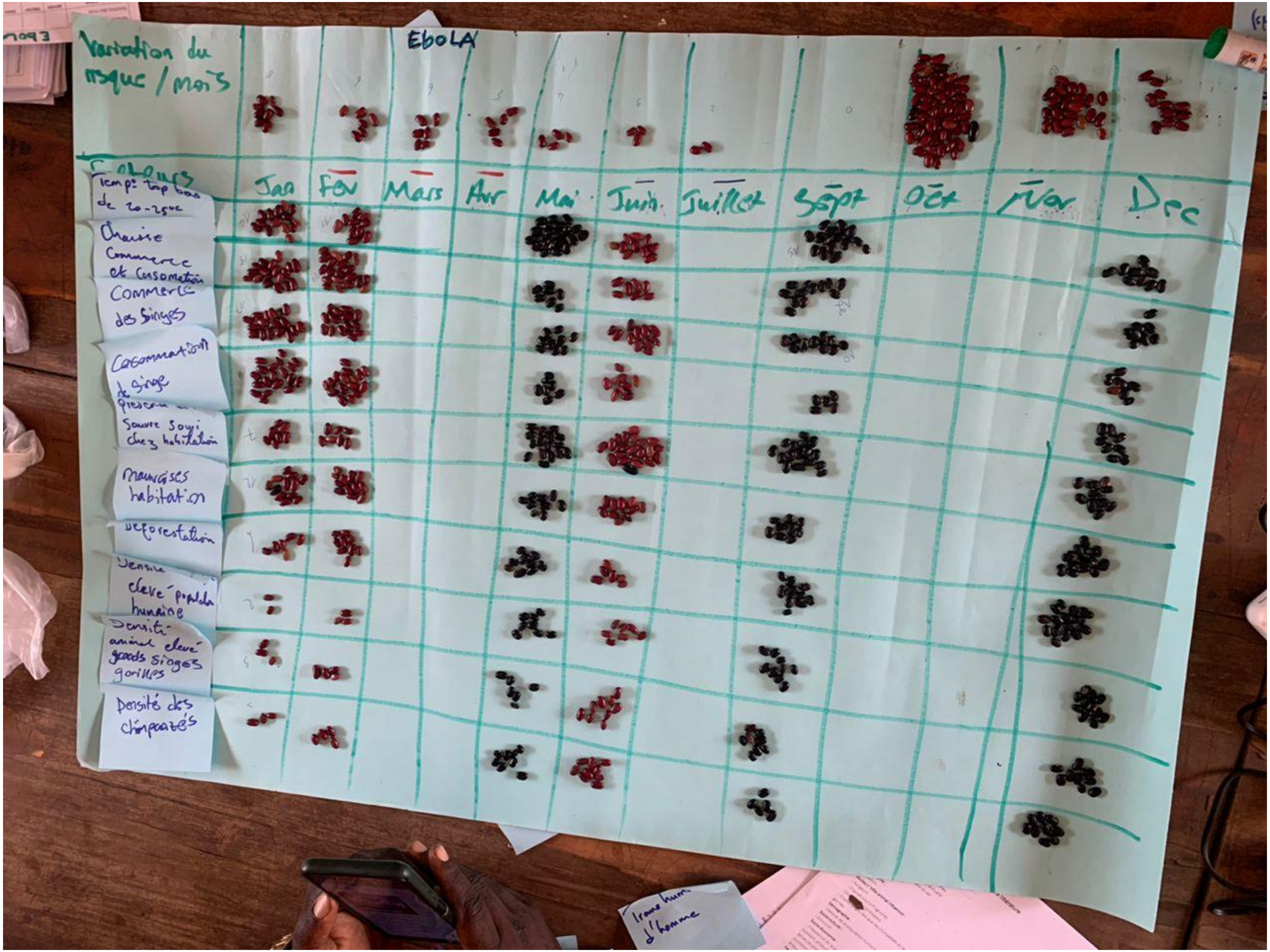
Variation du risque / Mois
EBOLA
Jan
Fév
Mars
Avr
Mai
Juin
Juillet
Sept
Oct
Nov
Dec
Chasse Commerce et Consomation
Commerce des Singes
Consommation de singe
Mauvaises habitation
Déforestation
Densité des chimpanzés

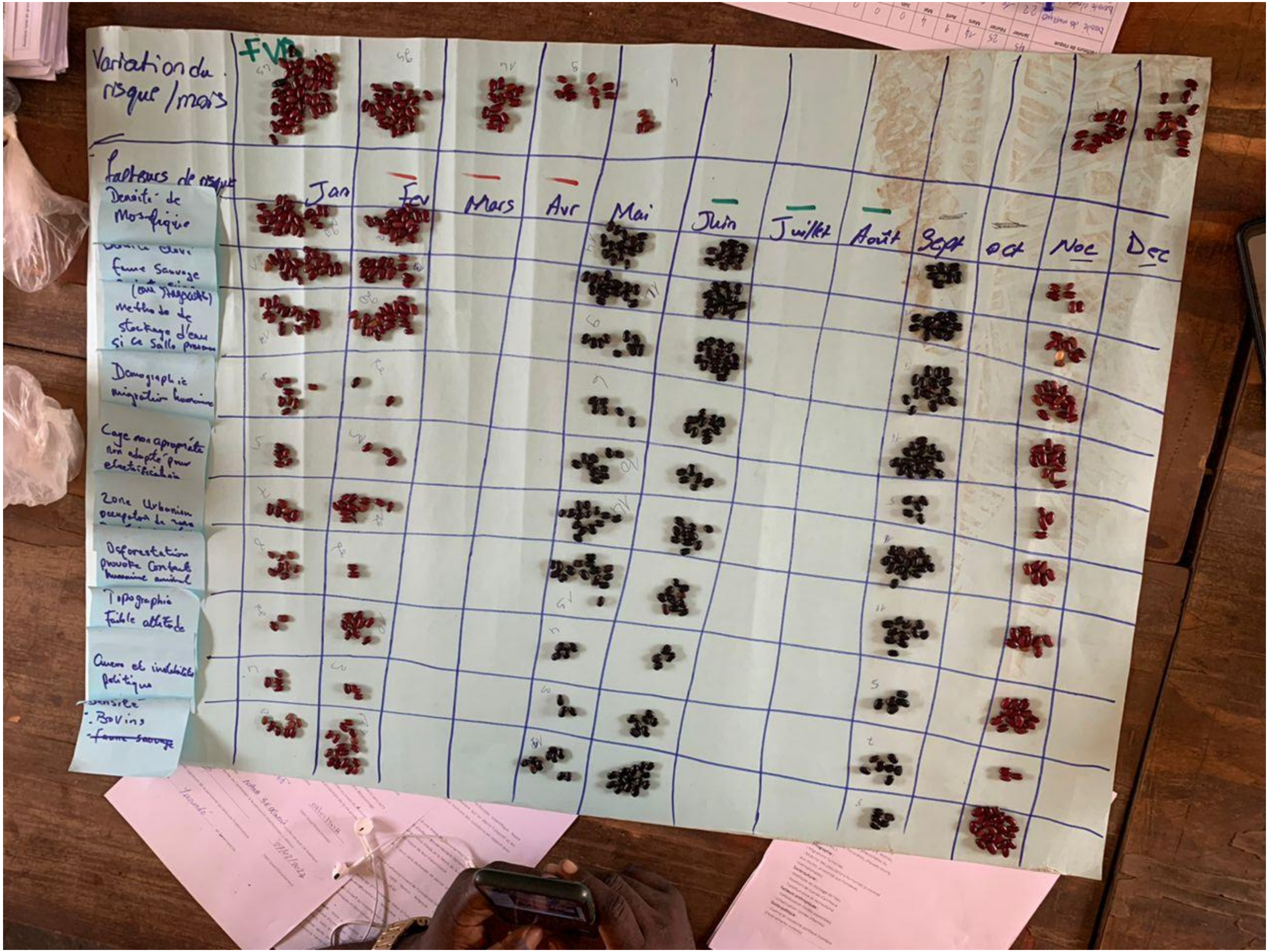
FVR
Variation du risque / mois
Facteurs de risque
Jan
Fev
Mars
Avr
Mai
Juin
Juillet
Aout
Sept
oct
Noc
Dec
Densité de Moustique
faune Sauvage
methode de stockage d'eau
Demographie migration humaine
Zone Urbanisation
Deforestation provoque Contact humaine animal
Topographie Faible altitude
Guerre et instabilité politique
Bovins
