# Supplementary material for: A participatory expert-based risk assessment to inform zoonotic disease surveillance in Cameroon: the case of Ebola virus disease and Rift Valley fever
Source: One Health Outlook. 2026 Aug 7;8:55. doi: 10.1186/s42522-026-00232-5 (PMC13732831; doi:10.1186/s42522-026-00232-5)
Supplement: Supplementary file 5 [file 42522_2026_232_MOESM6_ESM.pdf]

## RISK FACTORS DEFINITIONS

| **Risk factors** | **Definitions** |
|---|---|
| **Rainfall** | The quantity of rain falling within a given area in a given time |
| **El Nino Southern Oscillation (ENSO)** | A naturally occurring climate pattern with fluctuating ocean temperatures in the central and eastern Pacific, coupled with changes in the atmosphere |
| **Climate change** | A change in global or regional climate patterns, in particular a change apparent from the mid to late 20th century onwards and attributed largely to the increased levels of atmospheric carbon dioxide produced by the use of fossil fuels. |
| **Vegetation index** | A numerical representation, typically derived from remote sensing data, that assesses the presence, density, and health of vegetation in a given area |
| **Rainfed cropland** | Agricultural crops supplied with water by natural rainfall alone |
| **Pond** | Small artificial lake or retention basin that is stocked with fish and is used in aquaculture for fish farming, for recreational fishing, or for ornamental purposes |
| **Elevation** | The height of an object or point in relation to sea level |
| **Aedes density** | The concentration or abundance of Aedes mosquitoes in a specific area or space |
| **Transhumance** | A pastoral practice involving long-distance movement of animals, with the aim of overcoming the seasonal shortages in pasture availability |
| **Nomadism** | A way of life where people and their herds move from place to place, without a fixed settlement |
| **Livestock trade** | The buying and selling of cattle and sheep, most often in livestock markets. It includes the movement of these animals across national regions and international borders |
| **Cattle density** | The concentration or abundance of cattle in a particular area |
| **Sheep density** | The concentration or abundance of sheep in a particular area |
| **Zoological reserves** | Protected areas of land dedicated to the conservation of animal and plant life |
| **Wild ruminant density** | The concentration or abundance of wild ruminants in a specific area. These wild ruminants include buffalos (*Syncerus caffer*), giraffes (*Giraffa camelopardalis*), elands (*Taurotragus spp.*) and antelopes (*Kobus spp., Redunca spp., Gazella spp., Cephalophus spp., Hippotragus spp.*) |

| | |
|---|---|
| **Conflicts** | Socio-political crises leading to rebel takeovers and the displacement of local populations |
| **Saving water in small dams** | The practice of strategically storing and releasing water from smaller dams to ensure a consistent water supply, especially during times of drought or low water flow |
| **Small water bodies** | Permanent and temporary standing water bodies where animals can drink. They have a relatively small surface area compared to larger bodies like lakes or rivers. They include features like small lakes, marshes, floodplains, waterholes, and seasonal pools |
| **Goat density** | The concentration or abundance of goats in a particular area |
| **Irrigation** | The practice of supplying land with water so that crops and plants will grow |
| **Forest proximity** | The spatial closeness (often measured in meters or kilometres) of livestock areas to forest edges |
| **Deforestation** | The cutting down of trees in a large area, or the destruction of forests by people |
| **Urban areas** | Geographical spaces characterized by a high concentration of people and human-built structures like roads, sidewalks, and buildings. These typically include cities, towns, and their surrounding developed areas |
| **Temperature** | The measured amount of heat in a place |
| **Rivers** | Large natural streams of water flowing in a channel to the sea, a lake, or another river |
| **NHP density** | Non-Human Primates (monkeys, chimpanzees, gorillas) concentration or abundance in a particular area |
| **Bat density** | Concentration or abundance of bats in a particular area |
| **Antelope contact** | Hunting, live animal handling, meat processing, trade and consumption of antelopes |
| **Rodent contact** | Hunting, live animal handling, meat processing, trade and consumption of rodents |
| **Dogs and pigs densities** | Concentration or abundance of dogs and pigs in a particular area |
| **NHP hunting** | Hunting of monkeys, chimpanzees and gorillas |
| **Bat hunting** | Hunting or capture of bats |
| **Bushmeat hunting** | Hunting of NHP, bat, rodent and antelope |

| | |
|---|---|
| **NHP trade** | Any trading activities involving live and dead (meat) monkeys, chimpanzees and gorillas |
| **Bat trade** | Any trading activities involving live and dead (meat) bats |
| **Bushmeat trade** | Meat trade of NHP, bat, rodent and antelope |
| **NHP consumption** | Monkeys, chimpanzees and gorillas raw or cooked meat consumption |
| **Bat consumption** | Bat meat consumption |
| **Bushmeat consumption** | Meat consumption of NHP, bat, rodent and antelope |
| **NHP domestication** | Using monkeys and great apes as pets |
| **Human population density** | Number of people living within a specific area |
| **Socio-cultural practices** | Community-specific practices related to livelihood, forest-based subsistence, and behaviours that may shape human exposure to wildlife and bushmeat. |
| **Poverty** | The state of one who lacks a usual or socially acceptable amount of money or material possessions |
| **Education level** | The stages of formal education an individual has completed, typically categorized as primary, secondary, and higher education |
| **Forests** | Large areas of land covered with dense growth of trees and plants |
| **Cropland mosaics** | Landscapes composed of different types of fields, often interspersed with natural or semi-natural areas |
| **Evapotranspiration** | The process by which water is transferred from the land to the atmosphere by evaporation from the soil and other surfaces and by transpiration from plants. |
| **Drought** | Dry season: period when there is little or no rain |

NHP= Non-Human Primates
