# Supplementary material for: A participatory expert-based risk assessment to inform zoonotic disease surveillance in Cameroon: the case of Ebola virus disease and Rift Valley fever
Source: One Health Outlook. 2026 Aug 7;8:55. doi: 10.1186/s42522-026-00232-5 (PMC13732831; doi:10.1186/s42522-026-00232-5)
Supplement: Supplementary file 6 [file 42522_2026_232_MOESM7_ESM.pdf]

RIFT VALLEY FEVER

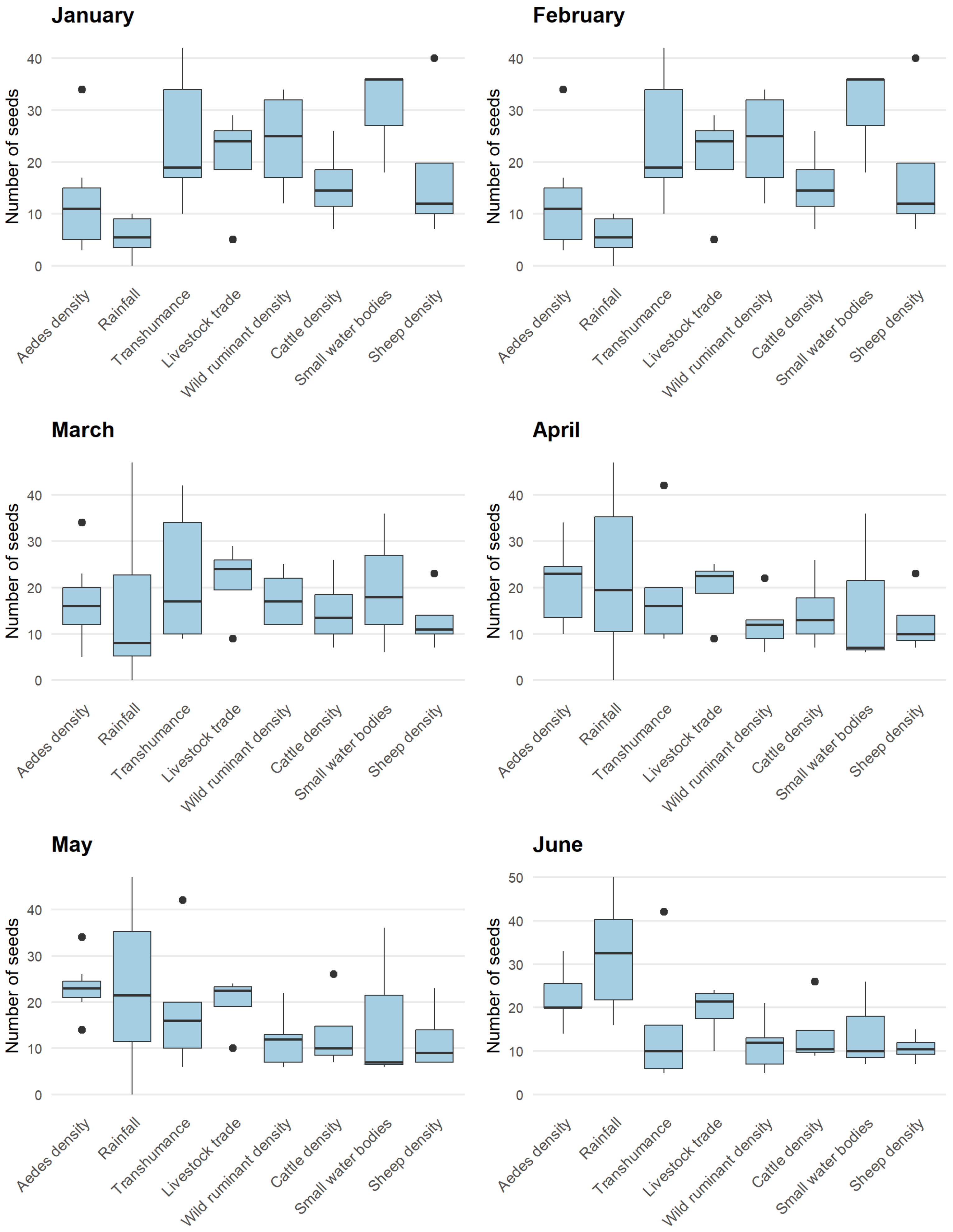

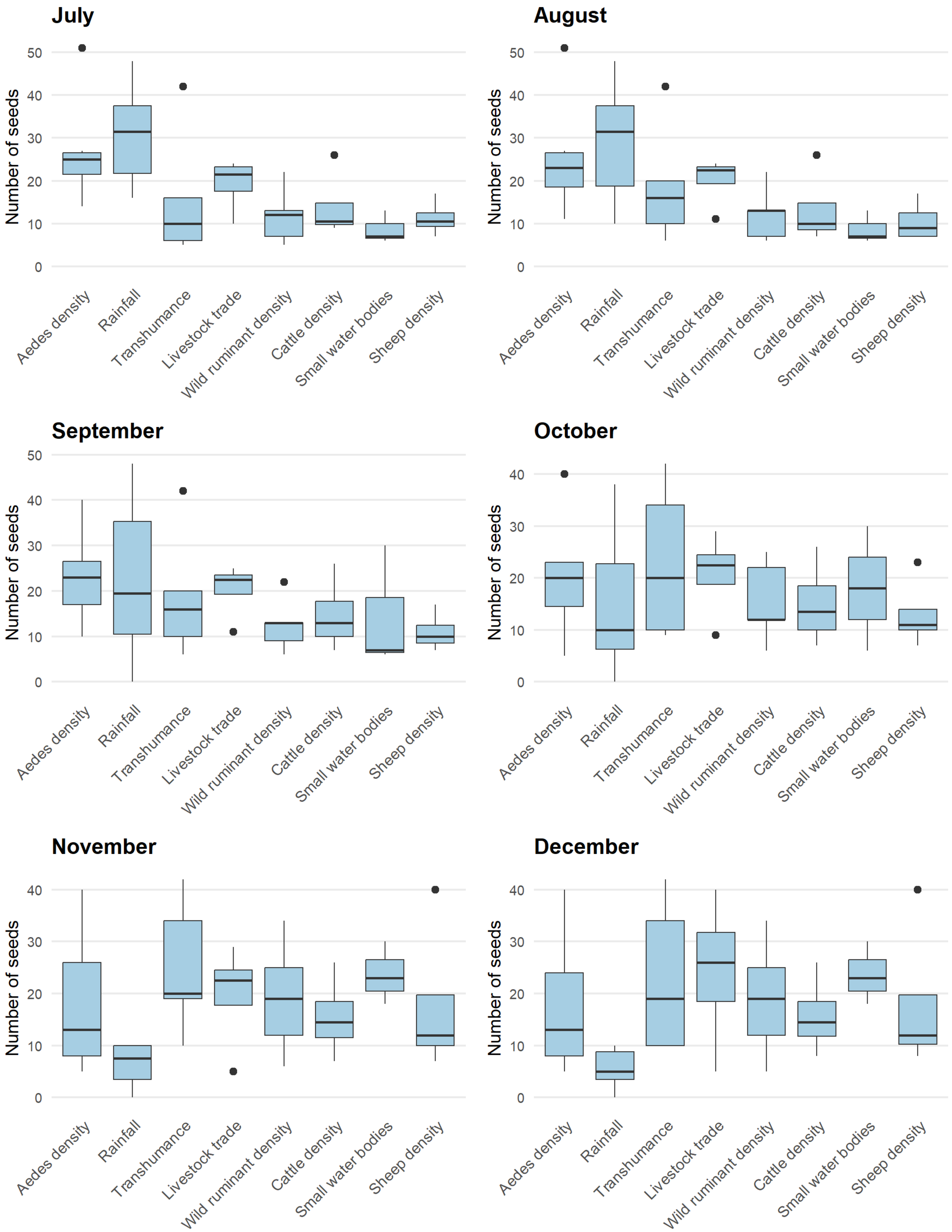
July
August
September
October
November
December
Number of seeds
Aedes density
Rainfall
Transhumance
Livestock trade
Wild ruminant density
Cattle density
Small water bodies
Sheep density
