# Supplementary material for: A participatory expert-based risk assessment to inform zoonotic disease surveillance in Cameroon: the case of Ebola virus disease and Rift Valley fever
Source: One Health Outlook. 2026 Aug 7;8:55. doi: 10.1186/s42522-026-00232-5 (PMC13732831; doi:10.1186/s42522-026-00232-5)
Supplement: Supplementary file 7 [file 42522_2026_232_MOESM8_ESM.pdf]

EBOLA VIRUS DISEASE

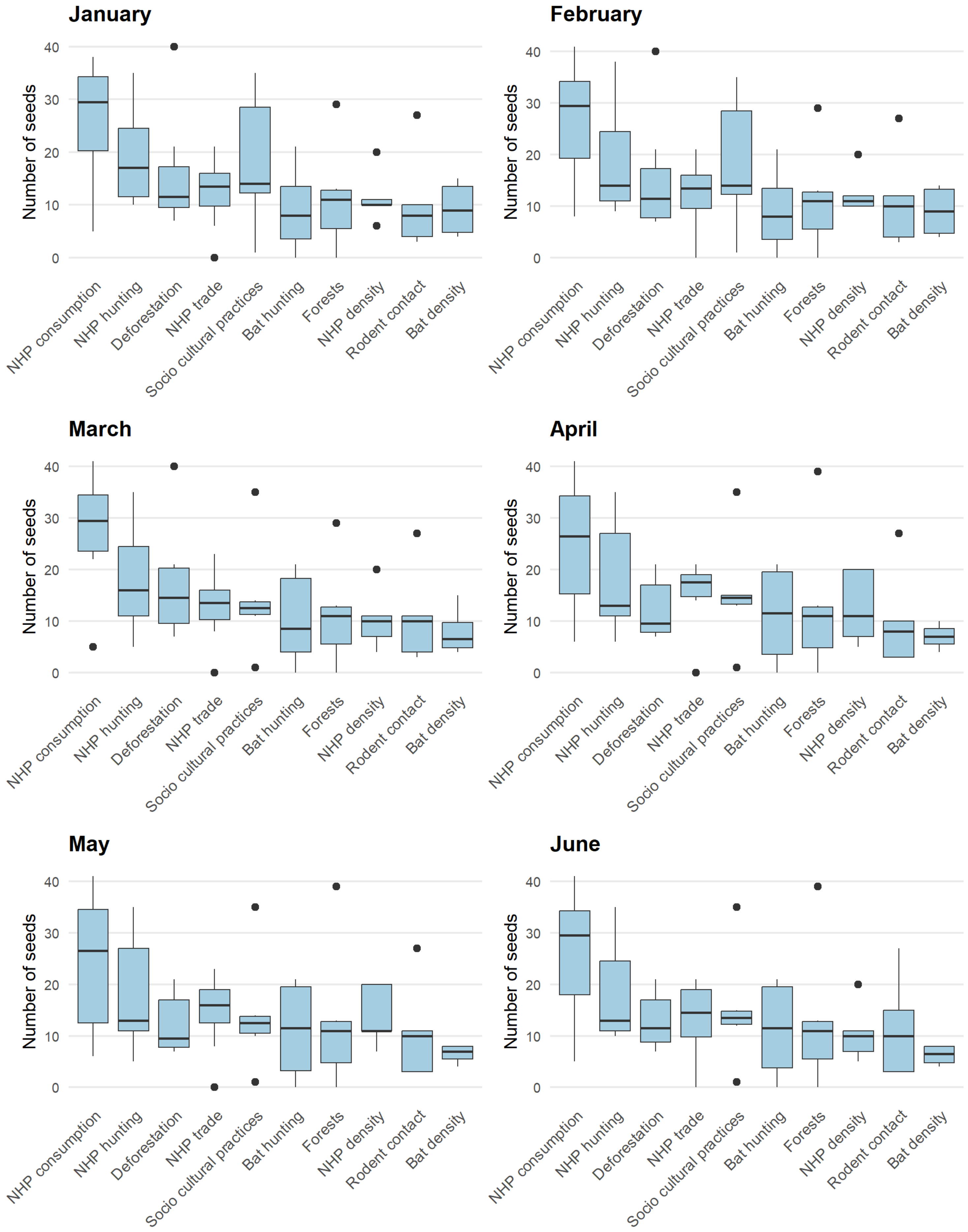

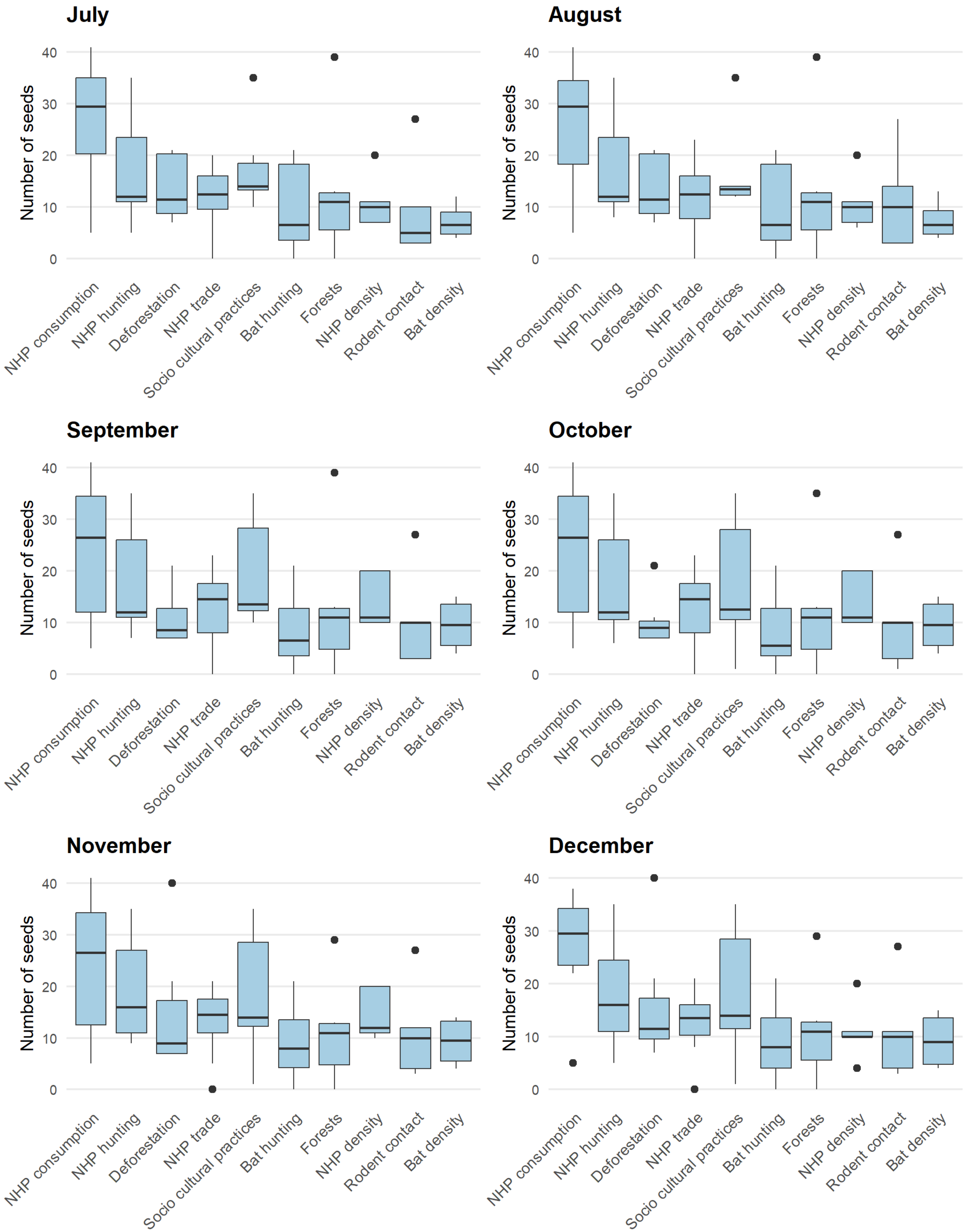
July
August
September
October
November
December
Number of seeds
0
10
20
30
40
NHP consumption
NHP hunting
Deforestation
NHP trade
Socio cultural practices
Bat hunting
Forests
NHP density
Rodent contact
Bat density
