# Supplementary material for: A participatory expert-based risk assessment to inform zoonotic disease surveillance in Cameroon: the case of Ebola virus disease and Rift Valley fever
Source: One Health Outlook. 2026 Aug 7;8:55. doi: 10.1186/s42522-026-00232-5 (PMC13732831; doi:10.1186/s42522-026-00232-5)
Supplement: Supplementary file 8 [file 42522_2026_232_MOESM9_ESM.pdf]

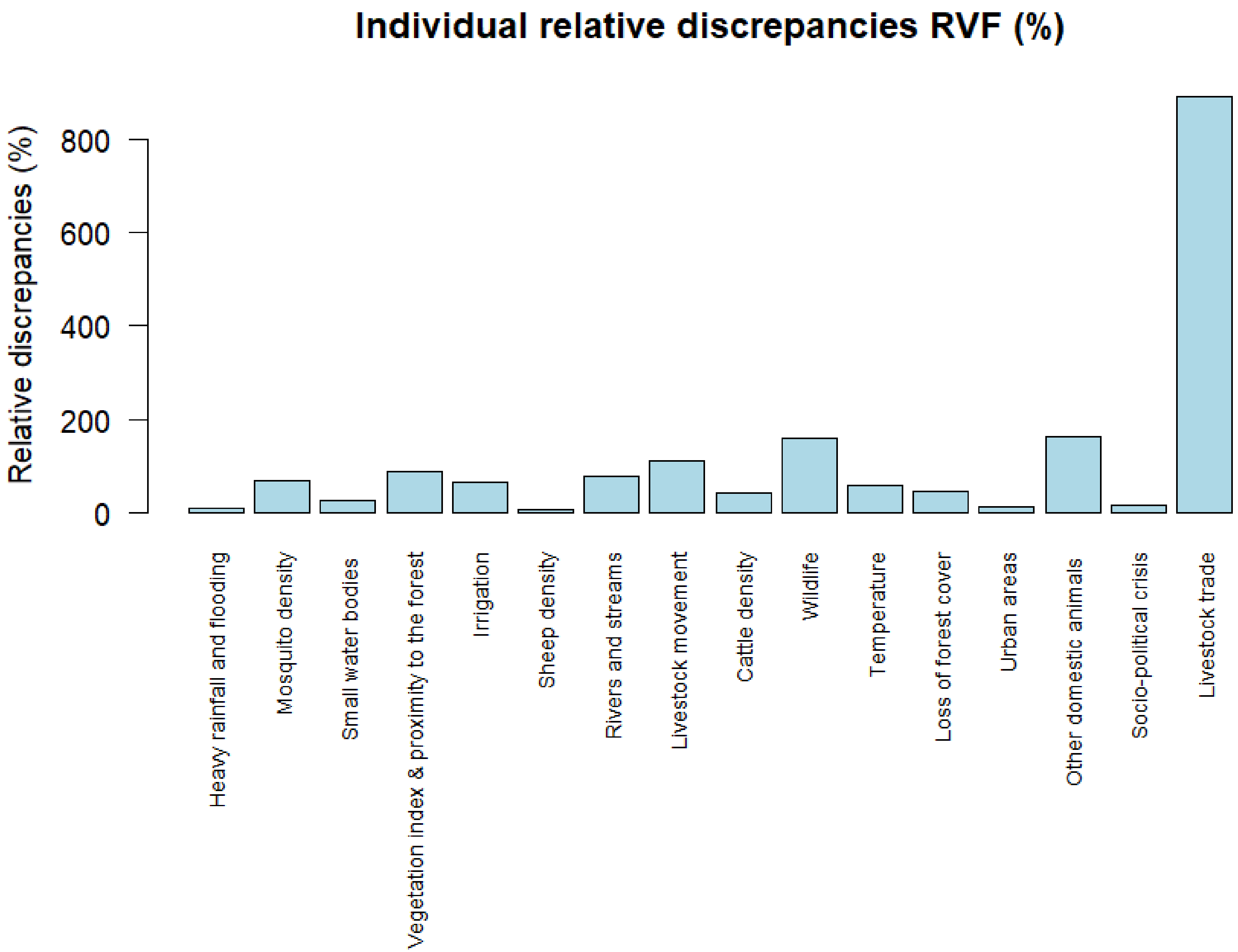

Individual relative discrepancies RVF (%)
Relative discrepancies (%)
0
200
400
600
800
Heavy rainfall and flooding
Mosquito density
Small water bodies
Vegetation index & proximity to the forest
Irrigation
Sheep density
Rivers and streams
Livestock movement
Cattle density
Wildlife
Temperature
Loss of forest cover
Urban areas
Other domestic animals
Socio-political crisis
Livestock trade
