# Supplementary material for: A participatory expert-based risk assessment to inform zoonotic disease surveillance in Cameroon: the case of Ebola virus disease and Rift Valley fever
Source: One Health Outlook. 2026 Aug 7;8:55. doi: 10.1186/s42522-026-00232-5 (PMC13732831; doi:10.1186/s42522-026-00232-5)
Supplement: Supplementary file 9 [file 42522_2026_232_MOESM4_ESM.docx]

**WEIGHTS FROM THE LITERATURE**

**RIFT VALLEY FEVER**

| **Literature Review Risk factors** | **No. Times associated** | **Total number of studies** | **Significance** | **References** |
| --- | --- | --- | --- | --- |
| Mosquitoes | 1+1+1+1+1+1+1+1+1+1 | 10 | *, NA, NA, NA, NA, NA, NA, NA, NA, NA | (Alhaji et al., 2020) (Nma Bida Alhaji et al., 2018) (Adamu et al., 2021) (Ateutchia Ngouanet et al., 2022) (Rissmann et al., 2020) (Danzetta et al., 2016) (Clements et al., 2006) (Lorenzo et al., 2015) (Rissmann et al., 2017) (Rodrigue Simonet et al., 2020) |
| heavy rainfall and flooding | 1+1+1+1+1+1+1+1+1+1+1+1 | 12 | *, NA, *, NA, NA, NA, NA, NA, NA, NA, *, NA | (Alhaji et al., 2020) (Nma Bida Alhaji et al., 2018) (Olaleye et al., 1996) (Bronsvoort et al., 2022) (Adamu et al., 2021) (Rodrigue Simonet et al., 2020) (Linthicum et al., 2016) (Danzetta et al., 2016) (Clements et al., 2006) (Nakouné et al., 2016)  (Canelas et al., 2022) (Rissmann et al., 2020) |
| Confirmed wildlife reservoirs for the RVF virus include African buffalos (*Syncerus caffer*), giraffes (*Giraffa camelopardalis*), desert warthogs (*Phacochoerus aethiopicus*), elephants (*Loxodonta africana*), rhinoceroses (*Diceros bicornis*), and *possibly bats* | 1+1+1+1 | 4 | NA, *, NA, NA | (Alhaji et al., 2018) (Bronsvoort et al., 2022) (Rissmann et al., 2020)  (Rodrigue Simonet et al., 2020) |
| Animal movement | 1+1+1+1+1+1 | 6 | *, NA, *, NA, NA, NA | (Alhaji et al., 2020) (Halawi et al., 2019) (N.B. Alhaji et al., 2018)(Rodrigue Simonet et al., 2020) (Rissmann et al., 2020) (Clements et al., 2006) |
| High cattle concentration | 1+1+1+1+1 | 5 | *, NA, NA, NA, NA | (Alhaji et al., 2020) (Nma Bida Alhaji et al., 2018) (Bronsvoort et al., 2022) (Rissmann et al., 2020) (Clements et al., 2006) |
| Sheep (mix grazing with sheep, sheep density) | 1+1+1+1+1+1+1 | 7 | NA, NA, NA, NA, NA, NA, * | (Bronsvoort et al., 2022) (N.B. Alhaji et al., 2018) (Rissmann et al., 2020) (Clark et al., 2018) (Clements et al., 2006) (Wright et al., 2019) (Nakouné et al., 2016) |
| Animal trade | 1 | 1 | NA | (Halawi et al., 2019) |
| Various domestic vertebrate hosts (including small ruminants, cattle, dogs, cats, horses, donkeys, and poultry | 1 | 1 | NA | (Rodrigue Simonet et al., 2020) |
| Flood plains, grazing and watering points, proximity to water points | 1+1+1+1+1+1+1+1+1 | 9 | NA, NA, NA, *, NA, NA, *, NA, NA | (Alhaji et al., 2018) (Bronsvoort et al., 2022) (Adamu et al., 2021) (N.B. Alhaji et al., 2018) (Rodrigue Simonet et al., 2020) (Rissmann et al., 2020) (Poueme et al., 2019) (Clements et al., 2006) (Wright et al., 2019) |
| Irrigation | 1+1+1+1+1+1+1+1 | 8 | *, NA, NA, NA, NA, NA, NA, NA | (Alhaji et al., 2020) (Nma Bida Alhaji et al., 2018) (Adamu et al., 2021) (Ateutchia Ngouanet et al., 2022) (Rodrigue Simonet et al., 2020) (Rissmann et al., 2020) (Clements et al., 2006) (Poueme et al., 2019) |
| Rivers and streams, proximity to hydrographic or large water banks | 1+1+1+1+1+1 | 6 | *, NA, NA, *, NA, NA | (Alhaji et al., 2020) (Nma Bida Alhaji et al., 2018) (Bronsvoort et al., 2022) (Poueme et al., 2019) (Clements et al., 2006) (Wright et al., 2019) |
| Bushy vegetation (vegetation index) | 1+1+1+1+1 | 5 | *, NA, *, NA, NA | (Alhaji et al., 2020) (Nma Bida Alhaji et al., 2018) (Adamu et al., 2021) (Linthicum et al., 2016) (Clements et al., 2006) |
| Forest cover (harbouring wild reservoirs and stagnant water bodies) | 1+1+1 | 3 | *, NA, NA | (Halawi et al., 2019) (Olaleye et al., 1996) (Rissmann et al., 2020) |
| Proximity to the forest | 1 | 1 | NA | (Halawi et al., 2019) |
| Aquaculture ponds | 1+1+1 | 3 | NA, NA, NA | (Alhaji et al., 2018) (Ateutchia Ngouanet et al., 2022) (Rissmann et al., 2017) |
| Saving water in small dams | 1 | 1 | NA | (Olaleye et al., 1996) |
| Altitude | 1+1+1+1 | 4 | *, *, NA, * | (Bronsvoort et al., 2022) (Adamu et al., 2021) (Clements et al., 2006) (Canelas et al., 2022) |
| Temperature | 1+1+1 | 3 | *, NA, NA | (Bronsvoort et al., 2022) (Clements et al., 2006) (Rissmann et al., 2020) |
| Urban areas | 1+1 | 2 | *, * | (Adamu et al., 2021) (Canelas et al., 2022) |
| Rainfed croplands | 1 | 1 | * | (Adamu et al., 2021) |
| Deforestation | 1+1 | 2 | NA, NA | (Ateutchia Ngouanet et al., 2022) (Nakouné et al., 2016) |
| Climate change | 1 | 1 | NA | (Ateutchia Ngouanet et al., 2022) |
| El Ni ̃no Southern Oscillation (ENSO) | 1 | 1 | NA | (Linthicum et al., 2016) |
| Socio-political crisis | 1 | 1 | NA | (Nakouné et al., 2016) |

Some studies did not provide a significance value for the factors (NA). Significance of the risk factor is shown as significant:

*p < 0.05 or ≤ 33% of variation explained

**p < 0.01 or 33% to 66% of variation

***p< 0.001 or > 66% of variation explained

| **Risk factors** | **Total number of studies** |
| --- | --- |
| **Climatic factors** | |
| Temperature | 3 |
| Heavy rainfall and flooding | 12 |
| El Ni ̃no Southern Oscillation (ENSO) | 1 |
| Climate change | 1 |
| **Environmental factors** | |
| Forest cover & proximity to the forest | 4 |
| Loss of forest cover | 2 |
| Elevation | 4 |
| Ponds | 3 |
| Irrigation | 8 |
| Small water bodies (Flood plains, grazing and watering points, proximity to water points) | 9 |
| Rivers and streams, proximity to hydrographic or large water banks | 6 |
| Bushy vegetation (vegetation index) | 5 |
| Urban areas | 2 |
| Rainfed croplands | 1 |
| **Vectors, reservoir & hosts** | |
| Mosquitoes | 10 |
| Wildlife | 4 |
| Cattle | 5 |
| Sheep | 7 |
| Other domestic animals (including small ruminants, cattle, dogs, cats, horses, donkeys, and poultry) | 1 |
| Animal movement (transhumance & nomadism) | 6 |
| Animal trade | 1 |
| **Human variables** | |
| Socio political crisis | 1 |
| Saving water in small dams | 1 |
| **Total number of occurrences** | **97** |

| **Risk factors** | **Total number of studies** | **Weights** |
| --- | --- | --- |
| Heavy rainfall and flooding | 12 | 0,12371134 |
| Mosquitoes | 10 | 0,103092784 |
| Small water bodies (Flood plains, grazing and watering points, proximity to water points) | 9 | 0,092783505 |
| Bushy vegetation + Forest cover (vegetation index& proximity to the forest) | 9 | 0,092783505 |
| Irrigation | 8 | 0,082474227 |
| Sheep | 7 | 0,072164948 |
| Rivers and streams, proximity to hydrographic or large water banks | 6 | 0,06185567 |
| Animal movement | 6 | 0,06185567 |
| Cattle | 5 | 0,051546392 |
| Elevation | 4 | 0,041237113 |
| Wildlife | 4 | 0,041237113 |
| Temperature | 3 | 0,030927835 |
| Ponds | 3 | 0,030927835 |
| Loss of forest cover | 2 | 0,020618557 |
| Urban areas | 2 | 0,020618557 |
| Rainfed croplands | 1 | 0,010309278 |
| Other domestic animals (including small ruminants, cattle, dogs, cats, horses, donkeys, and poultry) | 1 | 0,010309278 |
| Socio political crisis | 1 | 0,010309278 |
| Animal trade | 1 | 0,010309278 |
| Saving water in small dams | 1 | 0,010309278 |
| El Ni ̃no Southern Oscillation (ENSO) | 1 | 0,010309278 |
| Climate change | 1 | 0,010309278 |
| **Total** | **97** | **1** |

**EBOLA VIRUS DISEASE**

| **Literature Review Risk factors** | **No. Times associated** | **Total number of studies** | **Significance** | **References** |
| --- | --- | --- | --- | --- |
| Bushmeat hunting | 1+1+1+1+1+1+1+1+1+1+1 | 11 | NA, *, NA, NA, NA, NA, NA, NA, NA, NA, NA | (Wirsiy et al., 2021) (Mulangu et al., 2018) (LaBrunda and Amin, 2020) (Changula et al., 2014) (Lee-Cruz et al., 2021) (Kamorudeen et al., 2020) (Baseler et al., 2017) (Pigott et al., 2014) (Moyen et al., 2015) (Becquart et al., 2010) (Johnson et al., 1993) |
| Deforestation, forest fragmentation, logging activities | 1+1+1+1+1 | 5 | NA, NA, NA, NA, NA | (Kamorudeen et al., 2020) (Kock et al., 2019, pp. 2013–2016) (Gale et al., 2016) (Changula et al., 2014) (Groseth et al., 2007) |
| Ethnic background | 1+1+1 | 3 | *, NA, NA | (Johnson et al., 1993) (Wirsiy et al., 2021) (Groseth et al., 2007) |
| Bushmeat consumption (fruit bats, chimpanzees, gorillas, bush pig, monkeys, forest antelope and porcupines) | 1+1+1+1+1+1+1+1+1 | 9 | NA, NA, NA, NA, NA, *, NA, NA, NA | (Wirsiy et al., 2021) (Changula et al., 2014) (Lee-Cruz et al., 2021) (Baseler et al., 2017) (Pourrut et al., 2005) (Bratcher et al., 2022)  (Moyen et al., 2015) (Becquart et al., 2010) (Gale et al., 2016) |
| Contact with bats (bat hunting, consumption and during fruit harvesting) | 1+1+1+1+1+1 | 6 | ***, NA, NA, NA, NA, NA | (Moyen et al., 2015) (Becquart et al., 2010) (Lee-Cruz et al., 2021) (Gale et al., 2016) (Pourrut et al., 2005) (Bratcher et al., 2022) |
| Chimpanzee | 1+1+1+1+1+1 | 6 | NA, NA, NA, NA, NA, NA | (Pourrut et al., 2005)(E. M. Leroy et al., 2004) (Judson et al., 2016) (Wirsiy et al., 2021) (Changula et al., 2014) (Baseler et al., 2017) |
| Forests (proximity to forests, terra firme rainforests, vegetation, Enhanced Vegetation Index) | 1+1+1+1+1+1+1+1+1 | 9 | ***, *, NA, NA, NA, **, **, NA, *** | (Becquart et al., 2010) (Nkoghe et al., 2011) (Lee-Cruz et al., 2021) (Kamorudeen et al., 2020) (Kock et al., 2019, pp. 2013–2016)(Olivero et al., 2017) (Pigott et al., 2014) (Gale et al., 2016) (Walsh and Haseeb, 2015) |
| Bushmeat trade | 1+1+1+1+1 | 5 | NA, NA, NA, NA, NA | (LaBrunda and Amin, 2020) (Changula et al., 2014) (Lee-Cruz et al., 2021) (Moyen et al., 2015) (Becquart et al., 2010) |
| Gorilla | 1+1+1+1+1 | 5 | NA, NA, NA, NA, NA | (Pourrut et al., 2005) (Eric M. Leroy et al., 2004) (Redding et al., 2019) (E. M. Leroy et al., 2004) (Olivero et al., 2017) |
| Wildlife hosts (especially wild-borne chimpanzees) | 1+1+1+1 | 4 | NA, NA, *, NA | (Redding et al., 2019) (Gonzalez et al., 2018) (Olivero et al., 2017) (E. M. Leroy et al., 2004) |
| Bat distribution (reservoir) | 1+1 | 2 | NA, * | (Kock et al., 2019, pp. 2013–2016) (Pigott et al., 2014) |
| Poverty | 1+1+1+1+1+1+1 | 7 | NA, NA, NA, NA, NA, NA, NA | (Wirsiy et al., 2021) (LaBrunda and Amin, 2020) (Redding et al., 2019) (Weyer et al., 2015) (Shears and O’Dempsey, 2015)(Changula et al., 2014) (Kamorudeen et al., 2020) |
| Education level | 1+1 | 2 | NA, * | (Wirsiy et al., 2021) (Suchar et al., 2018) |
| War & conflicts | 1+1+1 | 3 | *, NA, NA | (Adegboye et al., 2021) (LaBrunda and Amin, 2020) (Weyer et al., 2015) |
| Contact with rodents | 1+1+1 | 3 | *, *, NA | (Mulangu et al., 2018) (Bratcher et al., 2022) (Olivero et al., 2017) |
| Contact with duikers | 1+1+1+1+1 | 5 | *, NA, NA, NA, NA | (Mulangu et al., 2018) (Pourrut et al., 2005) (Eric M. Leroy et al., 2004) (Redding et al., 2019) (Olivero et al., 2017) |
| Dogs and pigs | 1 | 1 | NA | (Changula et al., 2014) |
| High human population density | 1+1+1+1+1+1+1+1 | 8 | NA, NA, *, **, NA, NA, ***, NA | (Redding et al., 2019) (Lee-Cruz et al., 2021) (Suchar et al., 2018) (Olivero et al., 2017) (Baseler et al., 2017) (Schmidt et al., 2017) (Walsh and Haseeb, 2015) (Pigott et al., 2014) |
| Temperature Annual Range | 1+1+1+1 | 4 | **, NA, *, NA | (Redding et al., 2019) (Kock et al., 2019, pp. 2013–2016) (Suchar et al., 2018) (Amegah et al., 2016) |
| Mean Diurnal Temperature Range | 1+1+1+1+1 | 5 | *, NA, *, NA, *** | (Redding et al., 2019) (Kock et al., 2019, pp. 2013–2016) (Suchar et al., 2018) (Amegah et al., 2016) (Walsh and Haseeb, 2015) |
| Small annual temperature range | 1 | 1 | ** | (Olivero et al., 2017) |
| Night-time land surface temperature | 1 | 1 | * | (Pigott et al., 2014) |
| Precipitation of Wettest Month | 1+1 | 2 | **,* | (Redding et al., 2019) (Suchar et al., 2018) |
| Land Use-Land Cover (agricultural activities, cropland mosaics) | 1+1 | 2 | *, ** | (Redding et al., 2019) (Olivero et al., 2017) |
| Mean monthly rainfall | 1 | 1 | NA | (Lee-Cruz et al., 2021) |
| months of intermediate rainfall (100–250 mm) | 1 | 1 | NA | (Schmidt et al., 2017) |
| Elevation | 1+1+1 | 3 | NA, *, ** | (Kock et al., 2019, pp. 2013–2016) (Pigott et al., 2014) (Walsh and Haseeb, 2015) |
| Evapotranspiration | 1+1 | 2 | NA, * | (Kock et al., 2019, pp. 2013–2016) (Pigott et al., 2014) |

Some studies did not provide a significance value for the factors (NA). Significance of the risk factor is shown as significant:

*p < 0.05 or ≤ 33% of variation explained

**p < 0.01 or 33% to 66% of variation

***p< 0.001 or > 66% of variation explained

| **Risk factors** | **Total number of studies** |
| --- | --- |
| **Climatic factors** | |
| Mean Diurnal Temperature Range | 5 |
| Temperature Annual Range | 4 |
| Precipitation of Wettest Month | 2 |
| Evapotranspiration | 2 |
| Night-Time land surface temperature | 1 |
| Mean monthly rainfall | 1 |
| Months of intermediate rainfall (100–250 mm) | 1 |
| Small annual temperature range | 1 |
| **Environmental factors** | |
| Vegetation/forest cover | 9 |
| Loss of forest cover | 5 |
| Elevation | 3 |
| Cropland mosaics | 2 |
| **Human variables** | |
| Human population density | 8 |
| Poverty | 7 |
| Ethnic background | 3 |
| Education level | 2 |
| Bushmeat hunting | 11 |
| Bushmeat consumption | 9 |
| Bushmeat trade | 5 |
| **Wildlife reservoir & hosts** | |
| Bats | 8 |
| Monkeys | 7 |
| Chimpanzees | 6 |
| Gorillas | 5 |
| Duikers | 5 |
| Rodents | 3 |
| Dogs and pigs | 1 |
| **Total number of occurrences** | **116** |

| **Risk factors** | **Total number of studies** | **Weight** |
| --- | --- | --- |
| NHP density (Monkeys+ Gorillas+ Chimpanzees) | 18 | 0.1538462 |
| Bushmeat hunting (Monkeys+ Gorillas+ Chimpanzees +Bats+ Rodent+ Duikers) | 16 | 0.1367521 |
| Bushmeat consumption (Monkeys+ Gorillas+ Chimpanzees +Bats+ Rodent+ Duikers) | 12 | 0.1025641 |
| Temperature (Temperature Annual Range+ Mean Diurnal Temperature Range+ Night-time land surface temperature + Small annual temperature range) | 11 | 0.09482759 |
| Vegetation/forest cover | 9 | 0.07692308 |
| Human population density | 8 | 0.06837607 |
| Bat density | 8 | 0.06837607 |
| Poverty | 7 | 0.05982906 |
| Bushmeat trade | 5 | 0.04273504 |
| Loss of forest cover | 5 | 0.04273504 |
| Precipitation (Precipitation of Wettest Month + Mean monthly rainfall+ Months of intermediate rainfall (100–250 mm)) | 4 | 0.03418803 |
| Ethnic background | 3 | 0.02564103 |
| Elevation | 3 | 0.02564103 |
| Cropland mosaics | 2 | 0.01709402 |
| Education level | 2 | 0.01709402 |
| Evapotranspiration | 2 | 0.01709402 |
| Dogs and pigs | 1 | 0.008547009 |
| Total | 116 | 1 |

**References**

Adamu, A.M., Allam, L., Sackey, A.K.B., Nma, A.B., Mshelbwala, P.P., Mambula-Machunga, S., Idoko, S.I., Adikwu, A.A., Nafarnda, W.D., Garba, B.S., Owolodun, O.A., Dzikwi, A.A., Balogun, E.O., Simon, Y.A., 2021. Risk factors for Rift Valley fever virus seropositivity in one-humped camels (Camelus dromedarius) and pastoralist knowledge and practices in Northern Nigeria. One Health 13. https://doi.org/10.1016/j.onehlt.2021.100340

Adegboye, O., Gayawan, E., James, A., Adegboye, A., Elfaki, F., 2021. Bayesian spatial modelling of Ebola outbreaks in Democratic Republic of Congo through the INLA-SPDE approach. Zoonoses Public Health 68, 443–451. https://doi.org/10.1111/zph.12828

Alhaji, N.B., Aminu, J., Lawan, M.K., Babalobi, O.O., Ghali-Mohammed, I., Odetokun, I.A., 2020. Seropositivity and associated intrinsic and extrinsic factors for Rift Valley fever virus occurrence in pastoral herds of Nigeria: a cross sectional survey. BMC Vet. Res. 16, 243. https://doi.org/10.1186/s12917-020-02455-8

Alhaji, N.B., Babalobi, O.O., Isola, T.O., 2018. A quantitative exploration of nomadic pastoralists’ knowledge and practices towards Rift Valley fever in Niger State, North-central Nigeria: The associated socio-cultural drivers. One Health 6, 16–22. https://doi.org/10.1016/j.onehlt.2018.09.001

Alhaji, Nma Bida, Babalobi, O.O., Wungak, Y., Ularamu, H.G., 2018. Participatory survey of Rift Valley fever in nomadic pastoral communities of North-central Nigeria: The associated risk pathways and factors. PLoS Negl. Trop. Dis. 12, e0006858. https://doi.org/10.1371/journal.pntd.0006858

Amegah, A.K., Rezza, G., Jaakkola, J.J.K., 2016. Temperature-related morbidity and mortality in Sub-Saharan Africa: A systematic review of the empirical evidence. Environ. Int. 91, 133–149. https://doi.org/10.1016/j.envint.2016.02.027

Ateutchia Ngouanet, S., Wanji, S., Yadouleton, A., Demanou, M., Djouaka, R., Nanfack-Minkeu, F., 2022. Factors enhancing the transmission of mosquito-borne arboviruses in Africa. VirusDisease. https://doi.org/10.1007/s13337-022-00795-7

Baseler, L., Chertow, D.S., Johnson, K.M., Feldmann, H., Morens, D.M., 2017. The Pathogenesis of Ebola Virus Disease. Annu. Rev. Pathol. Mech. Dis. 12, 387–418. https://doi.org/10.1146/annurev-pathol-052016-100506

Becquart, P., Wauquier, N., Mahlakõiv, T., Nkoghe, D., Padilla, C., Souris, M., Ollomo, B., Gonzalez, J.-P., De Lamballerie, X., Kazanji, M., Leroy, E.M., 2010. High prevalence of both humoral and cellular immunity to Zaire ebolavirus among rural populations in Gabon. PloS One 5, e9126. https://doi.org/10.1371/journal.pone.0009126

Bratcher, A., Hoff, N.A., Doshi, R.H., Gadoth, A., Halbrook, M., Mukadi, P., Musene, K., Ilunga-Kebela, B., Spencer, D., Bramble, M.S., McIlwain, D., Kelly, J.D., Mukadi, D., Kingebeni, P.M., Ahuka, S., Okitolonda-Wemakoy, E., Muyembe-Tamfum, J.-J., Rimoin, A.W., 2022. Correction: Zoonotic risk factors associated with seroprevalence of Ebola virus GP antibodies in the absence of diagnosed Ebola virus disease in the Democratic Republic of Congo. PLoS Negl. Trop. Dis. 16. https://doi.org/10.1371/journal.pntd.0010465

Bronsvoort, B.M., Kelly, R.F., Freeman, E., Callaby, R., Bagninbom, J.M., Ndip, L., Handel, I.G., Tanya, V.N., Morgan, K.L., Ngwa, V.N., Rossi, G., Nfon, C.K., Mazeri, S., 2022. A Cross-Sectional, Population-Based, Seroepidemiological Study of Rift Valley Fever in Cameroonian Cattle Populations. Front. Vet. Sci. 9. https://doi.org/10.3389/fvets.2022.897481

Canelas, T., Thomsen, E., Kamgang, B., Kelly-Hope, L.A., 2022. Demographic and environmental factors associated with the distribution of Aedes albopictus in Cameroon. Med. Vet. Entomol. n/a. https://doi.org/10.1111/mve.12619

Changula, K., Kajihara, M., Mweene, A.S., Takada, A., 2014. Ebola and Marburg virus diseases in Africa: Increased risk of outbreaks in previously unaffected areas? Microbiol. Immunol. 58, 483–491. https://doi.org/10.1111/1348-0421.12181

Clark, M.H.A., Warimwe, G.M., Nardo, A.D., Lyons, N.A., Gubbins, S., 2018. Systematic literature review of Rift Valley fever virus seroprevalence in livestock, wildlife and humans in Africa from 1968 to 2016. PLoS Negl. Trop. Dis. 12, e0006627. https://doi.org/10.1371/journal.pntd.0006627

Clements, A.C., Pfeiffer, D.U., Martin, V., 2006. Application of knowledge-driven spatial modelling approaches and uncertainty management to a study of Rift Valley fever in Africa. Int. J. Health Geogr. 5, 57. https://doi.org/10.1186/1476-072X-5-57

Danzetta, M.L., Bruno, R., Sauro, F., Savini, L., Calistri, P., 2016. Rift Valley fever transmission dynamics described by compartmental models. Prev. Vet. Med. 134, 197–210. https://doi.org/10.1016/j.prevetmed.2016.09.007

Gale, P., Simons, R.R.L., Horigan, V., Snary, E.L., Fooks, A.R., Drew, T.W., 2016. The challenge of using experimental infectivity data in risk assessment for Ebola virus: why ecology may be important. J. Appl. Microbiol. 120, 17–28. https://doi.org/10.1111/jam.12973

Gonzalez, J.-P., Souris, M., Valdivia-Granda, W., 2018. Global spread of hemorrhagic fever viruses: Predicting pandemics. Methods Mol. Biol. 1604, 3–31. https://doi.org/10.1007/978-1-4939-6981-4_1

Groseth, A., Feldmann, H., Strong, J.E., 2007. The ecology of Ebola virus. Trends Microbiol. 15, 408–416. https://doi.org/10.1016/j.tim.2007.08.001

Halawi, A.-A.D., Saasa, N., Pongombo, B.L., Kajihara, M., Chambaro, H.M., Hity, M., Sawa, H., Takada, A., Mweene, A.S., Nsembo, L.L., Simulundu, E., 2019. Seroprevalence of Rift Valley fever in cattle of smallholder farmers in Kwilu Province in the Democratic Republic of Congo. Trop. Anim. Health Prod. 51, 2619–2627. https://doi.org/10.1007/s11250-019-01978-z

Johnson, E.D., Gonzalez, J.P., Georges, A., 1993. Filovirus activity among selected ethnic groups inhabiting the tropical forest of equatorial Africa. Trans. R. Soc. Trop. Med. Hyg. 87, 536–538. https://doi.org/10.1016/0035-9203(93)90077-4

Kamorudeen, R.T., Adedokun, K.A., Olarinmoye, A.O., 2020. Ebola outbreak in West Africa, 2014 – 2016: Epidemic timeline, differential diagnoses, determining factors, and lessons for future response. J. Infect. Public Health 13, 956–962. https://doi.org/10.1016/j.jiph.2020.03.014

Kock, R.A., Begovoeva, M., Ansumana, R., Suluku, R., 2019. Searching for the source of Ebola: the elusive factors driving its spillover into humans during the West African outbreak of 2013-2016. OIE Rev. Sci. Tech., Successes and remaining challenges within the One Health approach. 38, 113–122. https://doi.org/10.20506/rst.38.1.2946

LaBrunda, M., Amin, N., 2020. The Emerging Threat of Ebola. Adv. Sci. Technol. Secur. Appl. 103–139. https://doi.org/10.1007/978-3-030-23491-1_6

Lee-Cruz, L., Lenormand, M., Cappelle, J., Caron, A., De Nys, H., Peeters, M., Bourgarel, M., Roger, F., Tran, A., 2021. Mapping of Ebola virus spillover: Suitability and seasonal variability at the landscape scale. PLoS Negl. Trop. Dis. 15, e0009683. https://doi.org/10.1371/journal.pntd.0009683

Leroy, Eric M., Rouquet, P., Formenty, P., Souquière, S., Kilbourne, A., Froment, J.-M., Bermejo, M., Smit, S., Karesh, W., Swanepoel, R., Zaki, S.R., Rollin, P.E., 2004. Multiple Ebola Virus Transmission Events and Rapid Decline of Central African Wildlife. Science 303, 387–390. https://doi.org/10.1126/science.1092528

Leroy, E. M., Telfer, P., Kumulungui, B., Yaba, P., Rouquet, P., Roques, P., Gonzalez, J.-P., Ksiazek, T.G., Rollin, P.E., Nerrienet, E., 2004. A Serological Survey of Ebola Virus Infection in Central African Nonhuman Primates. J. Infect. Dis. 190, 1895–1899. https://doi.org/10.1086/425421

Linthicum, K.J., Britch, S.C., Anyamba, A., 2016. Rift Valley fever: an emerging mosquito-borne disease. Annu Rev Entomol 61, 395–415.

Lorenzo, G., López-Gil, E., Warimwe, G.M., Brun, A., 2015. Understanding Rift Valley fever: Contributions of animal models to disease characterization and control. Mol. Immunol., Non-rodent animal models for immunology research 66, 78–88. https://doi.org/10.1016/j.molimm.2015.02.001

Moyen, N., Thirion, L., Emmerich, P., Dzia-Lepfoundzou, A., Richet, H., Boehmann, Y., Dimi, Y., Gallian, P., Gould, E.A., Günther, S., de Lamballerie, X., 2015. Risk Factors Associated with Ebola and Marburg Viruses Seroprevalence in Blood Donors in the Republic of Congo. PLoS Negl. Trop. Dis. 9, e0003833. https://doi.org/10.1371/journal.pntd.0003833

Mulangu, S., Alfonso, V.H., Hoff, N.A., Doshi, R.H., Mulembakani, P., Kisalu, N.K., Okitolonda-Wemakoy, E., Kebela, B.I., Marcus, H., Shiloach, J., Phue, J.-N., Wright, L.L., Muyembe-Tamfum, J.-J., Sullivan, N.J., Rimoin, A.W., 2018. Serologic Evidence of Ebolavirus Infection in a Population With No History of Outbreaks in the Democratic Republic of the Congo. J. Infect. Dis. 217, 529–537. https://doi.org/10.1093/infdis/jix619

Nakouné, E., Kamgang, B., Berthet, N., Manirakiza, A., Kazanji, M., 2016. Rift Valley Fever Virus Circulating among Ruminants, Mosquitoes and Humans in the Central African Republic. PLoS Negl. Trop. Dis. 10, e0005082. https://doi.org/10.1371/journal.pntd.0005082

Nkoghe, D., Padilla, C., Becquart, P., Wauquier, N., Moussavou, G., Akué, J.P., Ollomo, B., Pourrut, X., Souris, M., Kazanji, M., Gonzalez, J.-P., Leroy, E., 2011. Risk Factors for Zaireebolavirus–Specific IgG in Rural Gabonese Populations. J. Infect. Dis. 204, S768–S775. https://doi.org/10.1093/infdis/jir344

Olaleye, O.D., Tomori, O., Ladipo, M.A., Schmitz, H., 1996. Rift Valley fever in Nigeria: infections in humans. Rev. Sci. Tech. Int. Off. Epizoot. 15, 923–935. https://doi.org/10.20506/rst.15.3.967

Olivero, J., Fa, J.E., Real, R., Farfán, M.Á., Márquez, A.L., Vargas, J.M., Gonzalez, J.P., Cunningham, A.A., Nasi, R., 2017. Mammalian biogeography and the Ebola virus in Africa. Mammal Rev. 47, 24–37. https://doi.org/10.1111/mam.12074

Pigott, D.M., Golding, N., Mylne, A., Huang, Z., Henry, A.J., Weiss, D.J., Brady, O.J., Kraemer, M.U., Smith, D.L., Moyes, C.L., Bhatt, S., Gething, P.W., Horby, P.W., Bogoch, I.I., Brownstein, J.S., Mekaru, S.R., Tatem, A.J., Khan, K., Hay, S.I., 2014. Mapping the zoonotic niche of Ebola virus disease in Africa. eLife 3, e04395. https://doi.org/10.7554/eLife.04395

Poueme, R., Stoek, F., Nloga, N., Awah-Ndukum, J., Rissmann, M., Schulz, A., Wade, A., Kouamo, J., Moctar, M., Eisenbarth, A., God-yang, L., Dickmu, S., Eiden, M., Groschup, M.H., 2019. Seroprevalence and Associated Risk Factors of Rift Valley Fever in Domestic Small Ruminants in the North Region of Cameroon. Vet. Med. Int. 2019, e8149897. https://doi.org/10.1155/2019/8149897

Pourrut, X., Kumulungui, B., Wittmann, T., Moussavou, G., Délicat, A., Yaba, P., Nkoghe, D., Gonzalez, J.-P., Leroy, E.M., 2005. The natural history of Ebola virus in Africa. Microbes Infect. 7, 1005–1014. https://doi.org/10.1016/j.micinf.2005.04.006

Redding, D.W., Atkinson, P.M., Cunningham, A.A., Lo Iacono, G., Moses, L.M., Wood, J.L.N., Jones, K.E., 2019. Impacts of environmental and socio-economic factors on emergence and epidemic potential of Ebola in Africa. Nat. Commun. 10. https://doi.org/10.1038/s41467-019-12499-6

Rissmann, M., Eiden, M., Wade, A., Poueme, R., Abdoulkadiri, S., Unger, H., Ziegler, U., Homeier, T., Groschup, M.H., 2017. Evidence for enzootic circulation of Rift Valley fever virus among livestock in Cameroon. Acta Trop. 172, 7–13. https://doi.org/10.1016/j.actatropica.2017.04.001

Rissmann, M., Stoek, F., Pickin, M.J., Groschup, M.H., 2020. Mechanisms of inter-epidemic maintenance of Rift Valley fever phlebovirus. Antiviral Res. 174, 104692. https://doi.org/10.1016/j.antiviral.2019.104692

Rodrigue Simonet, P.N., Alexandre Michel, N.-N., Abel, W., Albert, E., Martin Hermann, G., Franziska, S., 2020. Diversity and Abundance of Potential Vectors of Rift Valley Fever Virus in the North Region of Cameroon. Insects 11, 814. https://doi.org/10.3390/insects11110814

Schmidt, J.P., Park, A.W., Kramer, A.M., Han, B.A., Alexander, L.W., Drake, J.M., 2017. Spatiotemporal Fluctuations and Triggers of Ebola Virus Spillover - Volume 23, Number 3—March 2017 - Emerging Infectious Diseases journal - CDC. https://doi.org/10.3201/eid2303.160101

Shears, P., O’Dempsey, T.J.D., 2015. Ebola virus disease in Africa: Epidemiology and nosocomial transmission. J. Hosp. Infect. 90, 1–9. https://doi.org/10.1016/j.jhin.2015.01.002

Suchar, V.A., Aziz, N., Bowe, A., Burke, A., Wiest, M.M., 2018. An exploration of the spatiotemporal and demographic patterns of Ebola Virus Disease epidemic in West Africa using open access data sources. Appl. Geogr. 90, 272–281. https://doi.org/10.1016/j.apgeog.2017.10.003

Walsh, M.G., Haseeb, M.A., 2015. The landscape configuration of zoonotic transmission of Ebola virus disease in West and Central Africa: interaction between population density and vegetation cover. PeerJ 3, e735. https://doi.org/10.7717/peerj.735

Weyer, J., Grobbelaar, A., Blumberg, L., 2015. Ebola Virus Disease: History, Epidemiology and Outbreaks. Curr. Infect. Dis. Rep. 17, 1–8. https://doi.org/10.1007/s11908-015-0480-y

Wirsiy, F.S., Boock, A.U., Akoachere, J.-F.T.K., 2021. Assessing the determinants of Ebola virus disease transmission in Baka Community of the Tropical Rainforest of Cameroon. BMC Infect. Dis. 21, 324. https://doi.org/10.1186/s12879-021-06011-z

Wright, D., Kortekaas, J., Bowden, T.A., Warimwe, G.M., 2019. Rift Valley fever: biology and epidemiology. J. Gen. Virol. 100, 1187–1199. https://doi.org/10.1099/jgv.0.001296
